# Tethering of vesicles to the Golgi by GMAP210 controls LAT delivery to the immune synapse

**DOI:** 10.1038/s41467-019-10891-w

**Published:** 2019-06-28

**Authors:** Andres Ernesto Zucchetti, Laurence Bataille, Jean-Marie Carpier, Stéphanie Dogniaux, Mabel San Roman-Jouve, Mathieu Maurin, Michael W. Stuck, Rosa M. Rios, Cosima T. Baldari, Gregory J. Pazour, Claire Hivroz

**Affiliations:** 1 Institut Curie, PSL Research University, INSERM U932, Integrative analysis of T cell activation team, 26 rue d’Ulm, 75248 Paris Cedex 05, France; 2 Immunobiology Department, Yale University School of Medicine, New Haven, CT 06520 USA; 3 Program in Molecular Medicine, University of Massachusetts Medical School, Worcester, MA 01605 USA; 4 Cell Dynamics and Signaling Department, CABIMER-CSIC/US/UPO, 41092 Seville, Spain; 5 Department of Life Sciences, University of Siena, 53100 Siena, Italy

**Keywords:** Membrane trafficking, Lymphocyte activation, Imaging the immune system, T cells

## Abstract

The T cell immune synapse is a site of intense vesicular trafficking. Here we show that the golgin GMAP210, known to capture vesicles and organize membrane traffic at the Golgi, is involved in the vesicular transport of LAT to the immune synapse. Upon activation, more GMAP210 interact with LAT-containing vesicles and go together with LAT to the immune synapse. Regulating LAT recruitment and LAT-dependent signaling, GMAP210 controls T cell activation. Using a rerouting and capture assay, we show that GMAP210 captures VAMP7-decorated vesicles. Overexpressing different domains of GMAP210, we also show that GMAP210 allows their specific delivery to the immune synapse by tethering LAT-vesicles to the Golgi. Finally, in a model of ectopic expression of LAT in ciliated cells, we show that GMAP210 tethering activity controls the delivery of LAT to the cilium. Hence, our results reveal a function for the golgin GMAP210 conveying specific vesicles to the immune synapse.

## Abstract (web summary)

The immune synapse at the interface between T cells and antigen-presenting cells manifests intense vesicular trafficking, but how the vesicles are sorted is still unclear. Here the authors show that, in activated T cells, the Golgin GMAP210 specifically conveys LAT^+^ vesicles to immune synapse allowing proper T cell activation.

## Introduction

Communication of cells with their extracellular environment is a critical function for all eukaryotic cells. It is particularly important for the cells of the immune system that have to “sense” the danger in a full organism. This is probably why T lymphocytes have evolved to form a structure, specialized in cell–cell communication, which is generated upon direct contact between T lymphocytes and antigen-presenting cells and called the immune synapse^1,2^.

The immune synapse is a place of intense vesicular endocytic and exocytic traffic that control many aspects of T cell activities and functions. Indeed, the polarized release, at the immune synapse, of cytokines^3,4^, extracellular vesicles^5–7^, and receptors and ligands such as CD40L^8,9^, regulates their communication with the interacting cells and environment. It also shapes the adaptive immune response. This endocytic and exocytic traffic of vesicular compartments has also been shown to regulate TCR-induced signaling^10^. Recent results including ours have shown that molecules and enzymes, involved in signaling in T lymphocytes, are present both at the plasma membrane and in intracellular vesicular pools^11–17^. Remarkably different signaling mediators are present in different vesicles associated to a unique set of traffic regulators and effectors, such as Rab GTPase and v-SNARE proteins^10,18^. Hence different signaling molecules follow different endocytic and exocytic pathways. This may control the physical separation of signaling molecules in distinct modules that can be assembled in response to specific triggering^19^. These results also raise the question of the nature of the different vesicles, the control of their localization and of their polarized delivery to the immune synapse. We previously showed that trafficking to the immune synapse of the Linker for activation of T cells (LAT), a transmembrane protein that plays a key role in T lymphocyte signaling and function^20–23^, is regulated by the vesicular SNARE VAMP7^13^. We also recently found that the plasma membrane pool of LAT, after its TCR-induced internalization, is following the canonical retrograde Rab6/syntaxin 16 pathway to the Golgi before being transported back to the immune synapse^24^. This retrograde vesicular trafficking of LAT controls the formation of signaling complexes^13,24,25^, also known as signalosomes and regulates some aspects of T cell activation. Yet, the spatial organization of this traffic is not known. We describe herein a new mechanism by which a golgin, specifically conveys LAT-containing vesicles to the immune synapse.

Golgins are long coiled–coiled proteins attached to the Golgi membrane via their C-terminal part, which can “capture” vesicles at long distance through their N-terminal motifs increasing the efficiency of trafficking^26,27^. They have been proposed to ensure the specific delivery of vesicles containing given cargoes to the right membrane destination in the cells^26–29^. GMAP210 is one of these golgins. It anchors at the cis-Golgi via the interaction of its C-terminal GRAB (GRIP-related Arf binding) domain with Arf1^30–32^. It captures vesicles through its N-terminal domain^31^, which contains a curvature-sensing amphipathic lipid-packing sensor (ALPS) motif that binds liposomes of high membrane curvature (radius < 50 nm)^33^ and of particular lipid composition and packing^34^. GMAP210 has been involved in trafficking of Golgi resident membrane proteins and of ER to Golgi markers^28^. It has also been shown to bind the intraflagellar protein IFT20^35,36^ and controls trafficking of some cargos to the primary cilium and signaling by this structure^35,37,38^.

Here we identify the golgin GMAP210 as a specific binder of vesicles containing LAT/VAMP7. We show that it controls the polarized recruitment of LAT at the immune synapse, the formation of the LAT signalosome, and the TCR-induced activation of T lymphocytes. Our results reveal a mechanism that controls the correct localization of LAT to the immune synapse by tethering the VAMP7/LAT-positive vesicles to the Golgi. Hence our results reveal how the unique capacities of the golgin GMAP210 to capture and tether membranes are being used by T cells to selectively sort and deliver vesicles in the crowded environment of the immune synapse.

## Results

### Presence of GMAP210 in LAT-containing membranes

**Fig. 1:**
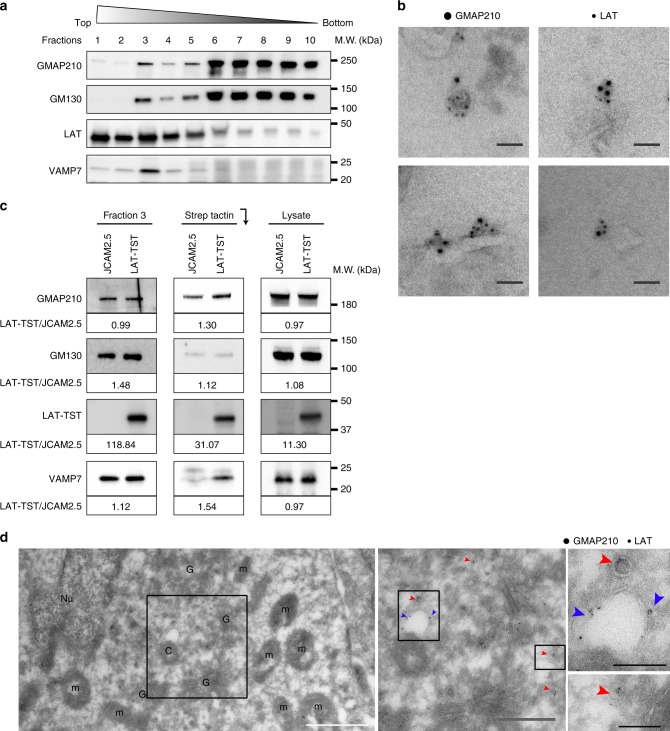
GMAP210 is present in membranes purified from T lymphocytes and containing LAT. **a** JCAM2.5 LAT-deficient T-cells expressing a chimeric mouse LAT coupled to two Strep-Tag (LAT-TST) were mechanically disrupted. The membrane fraction was then submitted to a floatation gradient on iodixanol. After ultracentrifugation, 10 fractions from top to bottom were collected and submitted to SDS–PAGE and Western blot analysis. **b** Transmission electron microscopy performed on membranes from fraction 3 showing an immunogold staining for LAT (6 nm gold particles) and GMAP210 (10 nm gold particles). Scale bar: 50 nm. **c** Fraction 3 prepared from JCAM2.5 (JCAM) or JCAM2.5 expressing LAT-TST (LAT-TST) were prepared as in **a**. They were mixed with Strep-Tactin Sepharose and submitted to SDS–PAGE and Western blot analysis. The presence of GMAP210, GM130, LAT-TST, and VAMP7 in: the fraction 3 before precipitation; the Strep-Tactin precipitates (StepTactin); and the total lysates obtained in the presence of detergent (Lysate), are shown. Ratios showing the relative expression of the different proteins in JCAM2.5 expressing LAT-TST as compared to the expression in JCAM2.5 are presented under each WB (LAT-TST/JCAM). **d** Transmission electron microscopy images of fixed Jurkat cells overexpressing LAT showing an immunogold staining for LAT (6 nm gold particles) and GMAP210 (10 nm gold particles); **c** centriole; **g** Golgi apparatus. Red arrows show small vesicles presenting both LAT and GMAP210 staining, blue arrows show a bigger vesicle “capping” a centriole. White Scale bar: 1 μm, gray scale bar: 500 nm, black scale bar: 200 nm. Data represent three independent experiments (**a**) and one experiment (**b**–**d**)

We have previously shown that LAT is present in vesicles that are recruited to the immune synapse^13^. To better characterize the content of these vesicles as well as their mechanisms of transport, we purified the vesicles containing LAT and performed a proteomic analysis of their content using a method we set up in the laboratory^39^. After mechanical disruption of LAT-deficient Jurkat T cells (JCAM-2.5)^40^ expressing the chimeric LAT-twin-Strep-Tag (LAT-TST)^25^, membranes were submitted to a floatation gradient. Fractions were recovered from top (fraction 1) to bottom (fraction 10). Fraction 3, which was enriched in both LAT and the v-SNARE VAMP7 involved in LAT trafficking^13^ (Fig. 1a), was submitted to pull down with Strep-Tactin Sepharose. The proteomic analysis of the purified material revealed the presence of the golgin GMAP210. Electron microscopy of fraction 3 confirmed the presence of GMAP210 and LAT on the same membranes (Fig. 1b). Western blot analysis performed on membranes from fraction 3 purified with Strep-Tactin Sepharose, confirmed the proteomic analysis. It showed an enrichment of GMAP210 in membranes from JCAM-2.5 expressing LAT-TST as compared to the control cells, whereas GMAP210 was present at the same level in fraction 3 and lysates of both cell types (Fig. 1c). GM130, another cis-golgin expressed by the cells (presence in the fraction 3 and whole lysates, Fig. 1c) was less present in the pull down (Fig. 1c), showing the specificity of the presence of GMAP210 together with LAT. Of note, VAMP7 was also enriched in the membranes purified from JCAM-2.5 cells expressing LAT-TST (Fig. 1c) confirming the presence of this vesicular SNARE on LAT-containing membranes^13^. Thus, GMAP210 is present together with LAT on intracellular membranes. This was surprising since GMAP210 was known to be present on the cis-Golgi, whereas the intracellular pool of LAT was mainly present in recycling compartments^11,15^. Electron microscopy performed on T cells showed that at steady state some LAT and GMAP210 were found together in small vesicles with a diameter inferior to 100 nm, located in the vicinity of the Golgi apparatus (Fig. 1d, red arrows). LAT and GMAP210 were also present together on the membrane of larger electron-translucent vesicles that seemed to “cap” one of the centrosomes (Fig. 1d, blue arrows) resembling the primary ciliary vesicle^41^. Our confocal microscopy analysis confirmed that in T lymphocytes, like in other cell types^31,42^, GMAP210 co-localized with GM130 and CTR-433 two markers of the cis-medial-Golgi but little with the Trans-Golgi network marker TGN-46 (supplementary Fig. 1).

### GMAP210 is recruited together with LAT to the immune synapse

**Fig. 2:**
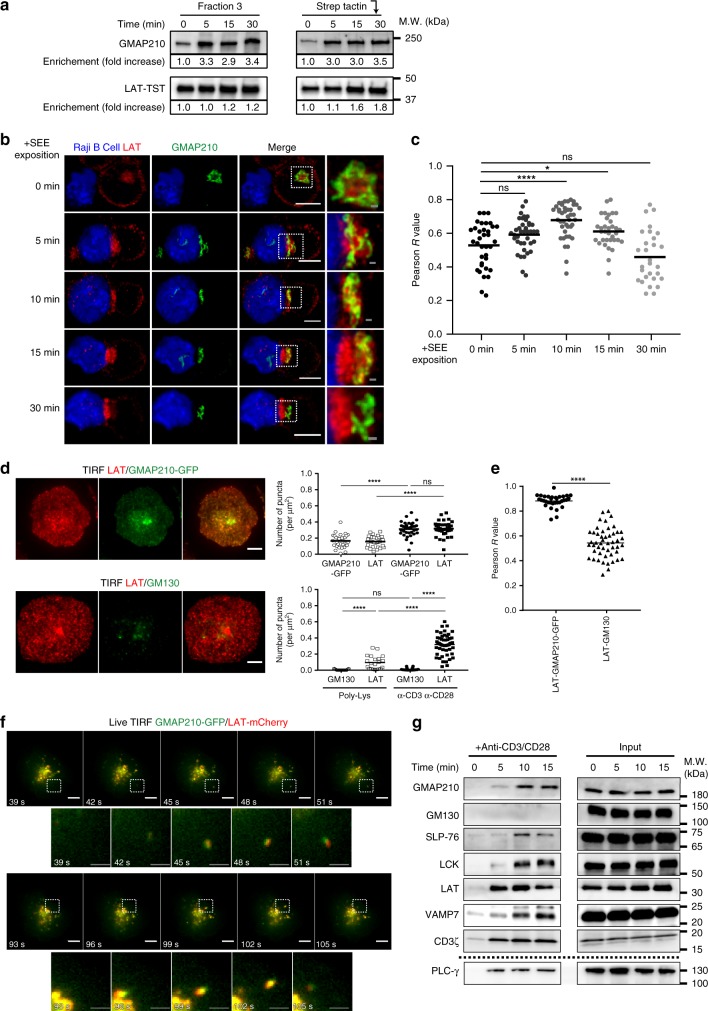
GMAP210 is recruited together with LAT at the immune synapse. **a** JCAM2.5 LAT-deficient T-cells expressing LAT-TST were activated for different time with anti-CD3ε+antiCD28, mechanically disrupted and membrane fractions were purified. Presence of GMAP210 and LAT-TST in fraction 3 and in precipitates (StepTactin), are shown. LAT-TST and GMAP210 intensities were quantified and expressed as fold increase of time 0. **b** Confocal images performed on Jurkat T-Raji conjugates (in blue) and pulsed with SEE for 0, 5, 10, 15 and 30 min, showing the relative localization of LAT and GMAP210. Images show the maximum intensity from z-projections of three–four z-stacks covering the GMAP210 staining. White scale bars: 5 μm, gray scale bars: 500 nm. **c** Quantification of GMAP210/LAT colocalization. Each dot = one cell; horizontal lines = median. *P < 0.05, ****P < 0.0001, ns = non-significant (one-way ANOVA). **d** TIRFM images of Jurkat cells incubated for 10 min on coverslips coated with anti-CD3ε+anti-CD28, before fixation and staining for LAT, GMAP210-GFP, or GM130, scale bars: 5 μm. Dot plots show the quantification of the number of punctas/µm^2^ formed by the different molecules in the evanescent field (right). Poly-l-Lysine (Poly-Lys) alone (resting conditions) or anti-CD3/CD28 (α-CD3ε α-CD28) immune synapse formation. **e** Quantification of the colocalization of LAT with GMAP210 or GM130. Each dot = one cell; horizontal lines = median. ****P < 0.0001, ns: non-significant (one-way ANOVA). **f** Live TIRF imaging of the recruitment of LAT and GMAP210 at the immune synapse. Jurkat cells co-expressing GMAP210-GFP and LAT-mCherry were seeded on coverslips coated with anti-CD3ε+antiCD28. White squares indicate the magnified regions presented underneath that show the simultaneous appearance and displacement of LAT and GMAP210 in the evanescent field. White scale bars: 5 μm, gray scale bars: 2 μm. **f** Immunoblot of signalosomes prepared from Jurkat cells activated for 0, 5, 10 or 15 min with magnetic beads coated with mAb to CD3 and to CD28 (above blots). Proteins attached to the beads were purified by magnetic sorting after freezing and thawing of the cells. Presence of the different proteins in the corresponding cell lysates (with detergent) are shown in “input” lanes. Dashed line indicates a separate experiment. Data represent more than three experiments (**g**), two experiments (**f**), and one experiment (**a**–**e**)

Previous results were obtained at steady state. We asked whether activation of T cells would regulate the presence of GMAP210 on LAT-containing vesicles. Fractionation experiments described above were performed on Jurkat T cells activated, for several times with anti-CD3 + anti-CD28. We first noticed that T cell activation induced GMAP210 enrichment in fraction 3 as compared to resting T cells (Fig. 2a). This was accompanied by a three-fold increase in the presence of GMAP210 on the LAT-TST-containing vesicles purified with streptactin (Fig. 2a). These results show that T cell activation induces the recruitment of GMAP210 on LAT-containing vesicles. They correlated with confocal images realized on conjugates between Jurkat T cells and Raji B cells used as antigen-presenting cells (APC). Indeed, at steady state, i.e. in the absence of SEE, the intracellular pool of LAT showed only inconspicuous colocalization with GMAP210. Activation with SEE induced the rapid recruitment of LAT at the immune synapse, which was accompanied in the first 15 min by a polarization of GMAP210 toward the synapse and an intertwined localization of both molecules (Fig. 2b and quantified in Fig. 2c). At 30 min GMAP210 was slightly behind the immune synapse. In these conditions, as reported by others^43,44^, we often observed the rims of the Golgi stacks, labeled with GMAP210, in close proximity to the area of the immune synapse where LAT was enriched (Fig. 2b). To study more precisely if GMAP210 was recruited to the synaptic zone together with LAT, we performed total internal reflection fluorescence microscopy (TIRFM) in T cells expressing GMAP210-GFP. Cells were seeded on activating coverslips coated with anti-CD3 and anti-CD28 mAbs, to mimic immune synapse formation, or with poly-l-Lysine as control (Fig. 2d). Quantification of the TIRFM images, performed 10 min after seeding, revealed “punctae” of GMAP210 that can be observed in activating conditions (Fig. 2d, upper panel and quantification on the right). These “punctae” were revealed by TIRFM, showing that they were present in a 200–300 nm of the contact zone. The density of GMAP210 and LAT punctae were very similar (Fig. 2d, quantification) and GMAP210 and LAT co-localized strongly (Fig. 2e, quantification). Of note, images were taken 10 min after interaction with the activating slide, at a time point shown by others to correspond to a recruitment of abundant vesicles^45^. Because GMAP210 was never observed in the plasma membrane, our results suggest that the punctae revealed by TIRFM correspond to vesicular pool of LAT coming together with GMAP210 in the synaptic zone (Fig. 2d). The co-localization of LAT with GMAP210 was not due to the mere recruitment of the Golgi apparatus to the immune synapse, since neither the cis-golgin GM130 (Fig. 2d, lower panel, co-localization quantified in 2e), nor the medial-Golgi marker CTR433^46^ (Supplementary Fig. 2a and quantified in Ssupplementary Fig. 2b) co-localized with LAT at the immune synapse. This was not an artifact of overexpression of GMAP210-GFP, since colocalization of LAT with endogeneous GMAP210 was also observed (Supplementary Fig. 2a, lower panels and quantification of colocalization Supplementary Fig. 2b). Live video microscopy of T cells expressing both LAT-mCherry and GMAP210-GFP seeded on activating coverslips showed that LAT and GMAP210 arrived at the same time in the evanescent field. They also moved together suggesting that GMAP210, which is not present at the plasma membrane, is associated with the vesicular pool of LAT and recruited to the inner face of the IS (Fig. 2f, Supplementary Movie 1). We have previously shown that the vesicular pool of LAT contributes to the formation of a signalosome^47^, which is assembled upon TCR triggering^13^. We reasoned that if GMAP210 is recruited together with LAT at the immune synapse, it might be part of this signalosome. To test this hypothesis, we activated T cells with magnetic beads coated with anti-CD3+CD28 mAb, retained the bead-cell conjugates on a magnet and subjected them to cycles of freezing and thawing. Western blot analysis of the bead-associated complexes revealed the presence of LAT, as well as different signaling molecules: the adaptor SLP76, the tyrosine kinase p56lck and the phospholipase PLCγ1 (Fig. 2f), which play a role in T cell activation. It also revealed the progressive recruitment of GMAP210 with the same kinetic as VAMP7 (Fig. 2g). The absence of GM130 from this signalosome demonstrated that the presence of GMAP210 was not due to a mere contamination by material from the Golgi apparatus.

Altogether these results suggest that TCR activation induces the rapid recruitment of GMAP210 on vesicles containing LAT and suggest a role for GMAP210 in the delivery of the vesicular pool of LAT.

### GMAP210 controls the delivery of LAT to the immune synapse

**Fig. 3:**
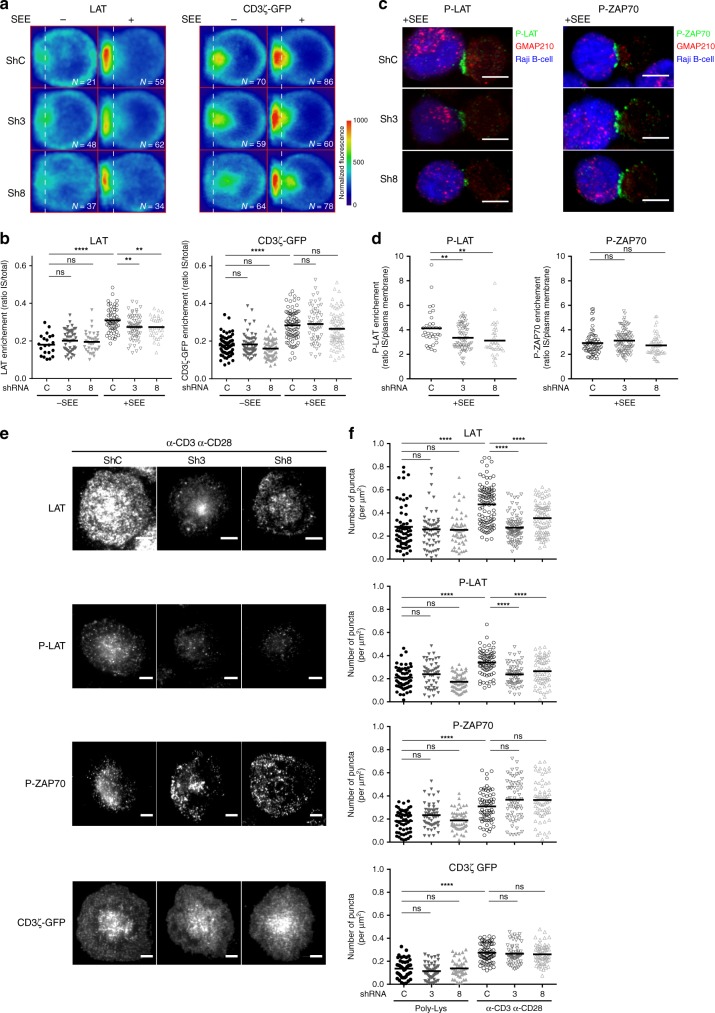
GMAP210 expression controls formation of the immune synapse. **a**, **b** Confocal images **a** and quantification **b** of the enrichment of LAT (left pannel) and CD3ζ-GFP (rigth pannel) at the immune synapse (depicted by the dotted white line) in Jurkat “mean cells” expressing non-targeting control ShRNA **c** or GMAP210-targeting ShRNA (3 and 8) and interacting for 30 min with Raji cells left unpulsed (−, unactivated state) or pulsed with SEE (+, immune synapse formation). *N* = number of cells constituting the mean image. Horizontal lines represent median. **c** and **d** Confocal images of conjugates of Jurkat cells expressing control (C) or GMAP210-specific shRNA (3 and 8) and SEE-pulsed Raji B cells (blue) labeled with anti-phospho LAT (P-LAT, showed in green, left pannel) or anti-phospho-ZAP70 (P-ZAP, showed in green, right pannel) and anti-GMAP210 (red) antibodies, assessed at 30 min **c**, and quantification **d** of the mean fluorescence intensity of P-LLAT and P-ZAP70, assessed in a fixed region of the immunimmune synapse and divided by the average of the mean intensities measured in three regions of the same size at the plasma membrane outside of the IS. Horizontal lines represent median. **e** TIRF images of endogenous LAT, P-LAT, P-ZAP70, or CD3ζ-GFP in Jurkat cells expressing non-targeting control ShRNA **c** or GMAP210-specific ShRNA (3, 8), incubated for 10 min on coverslips coated with poly-l-Lysine alone (resting conditions) or anti-CD3ε+antiCD28 Abs (α-CD3 α-CD28, activating conditions) before fixation and staining. **f** Quantification, in the evanescent field, of the density of the number of punctas of different proteins and phospho-proteins in Jurkat cells. Each dot = one cell; horizontal lines = median. Scale bars = 5 μm, **P < 0.01, ****P < 0.0001, ns: non-significant (one-way ANOVA). Data are from two independent experiments in **a**, **b**, **c**, and **d** and three independent experiments in **e** and **f**

**Fig. 4:**
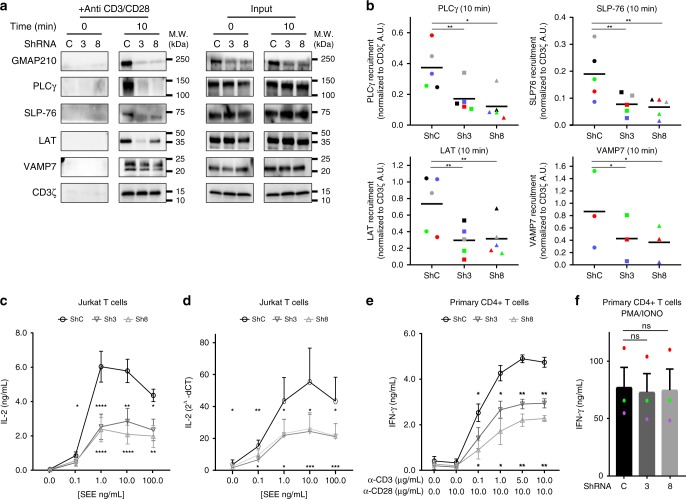
GMAP210 silencing inhibits activation of T lymphocytes. **a** Immunoblot analysis of signalosomes prepared from Jurkat cells expressing control (C) or GMAP210 specific ShRNA (3, 8) activated for 0, or 10 min with magnetic beads coated with mAb to CD3ε and to CD28 (above blots). Proteins attached to the beads were purified by magnetic sorting after freezing and thawing of the cells without detergent (anti-CD3/CD28). Presence of the different proteins in the corresponding cell lysates are shown in “input” lanes. **b** Quantification of PLCγ, SLP-76, LAT, and VAMP7 band intensities at 10 min of activation and normalized on CD3ζ intensity band. **c** and **e** Enzyme-linked immunosorbent assay of IL-2 in supernatants of Jurkat cells (**c**) or IFN-γ in human primary CD4+ T cells (**e**) expressing control (circle) or GMAP210-specific ShRNA (triangle) and activated for 6 h by Raji B cells pulsed with various concentrations (horizontal axis) of SEE (**c**, Jurkat T cells) or anti-CD3 in the presence of 10 μg/mL of anti-CD28 (**e**, human primary T cells). **d** Quantitative PCR analysis of IL-2 in Jurkat cells activated as in **c**). **f** Enzyme-linked immunosorbent assay of IFN-γ in supernatants of human primary CD4+ T cells expressing control (ShC) or GMAP210-specific ShRNA (Sh3 and Sh8) and activated for 6 h with a combination of PMA+ionomycin that bypasses LAT-signaling. *P < 0.05, **P < 0.01, ***P < 0.001, ****P < 0.0001, ns: non-significant (paired parametric *t*-tests, one tail). Mean is represented by horizontal lines in **b** and each experiment is represented by one color. Data represent between three and five experiments in **a**, **b**, **c,** and **e**, and three experiments in **d** and **f**

To test whether GMAP210 was involved in the recruitment of the vesicular pool of LAT to the immune synapse, we silenced GMAP210 in Jurkat T cells or human primary CD4^+^-activated T cells using lentivirus encoding either of two different short hairpin RNAs (shRNAs) targeting GMAP210 (Sh3 or Sh8). In both Jurkat and primary T cells, GMAP210 protein expression was decreased to 40% of the control cells (Supplementary Fig. 3a for Jurkat and Supplementary Fig. 3c for primary T cells). We controlled the expression at the plasma membrane of different key markers of T cells, such as CD3, CD28, TCR, CD4, and CD45, which was not affected by GMAP210 silencing (Supplementary Fig. 3b for Jurkat and Supplementary Fig. 3d for primary T cells) showing that GMAP210 silencing did not grossly affect secretion at the plasma membrane in T cells. Besides, GMAP210 silencing did not affect the expression of LAT in T cells. Indeed, silenced T cells expressed the same amount of LAT (Supplementary Fig. 3b for Jurkat and Supplementary Fig. 3d for primary T cells). Moreover, at steady state, expression of LAT at the plasma membrane, as measured by FACS on T cells expressing a chimeric LAT tagged with HA in its N-term extracellular region (HA-LAT)^13,24^ was not affected either (Supplementary Fig. 3b). We then quantified the recruitment of LAT to the immune synapse. To do so we first quantified LAT enrichment at the immune synapse in Jurkat T cells interacting with Raji B cells in the absence of SEE (no synapse formation) or presence of SEE (formation of the immune synapse) (average density map representation in a “mean cell” Fig. 3a and Supplementary Fig. 4a, and quantification in Figs. 3b and 4b). We also quantified on TIRFM images the number of LAT punctae in the synaptic area in Jurkat T cells or CD4^+^ human primary T cell blasts 10 min after seeding on activating slides (Fig. 3e, f for Jurkat and Supplementary Fig. 4c: quantification in primary T cells). In both models, LAT recruitment was decreased when GMAP210 was silenced (quantification Fig. 3b, d and Supplementary Fig. 4c). This defect in LAT recruitment at the immune synapse led to a decreased phosphorylation of LAT (Tyr-191) observed in conjugates (Fig. 3c representative images, quantification in Fig. 3d) and by TIRFM (for Jurkat Fig. 3e quantified in Fig. 3f; for primary T cells quantification in Supplementary Fig. 4c). These results suggested that the pool of LAT that is recruited in a GMAP210-dependent manner is phosphorylated. In contrast, recruitment of other signaling molecules, such as CD3-ζ, the phosphorylated form of ZAP70 (Fig. 3c–f for Jurkat T cells, Supplementary Fig. 4c for primary T cells) and the TCR (Supplementary Fig. 4a and 4b), which like LAT are enriched at the IS upon T-cell activation, was not affected by silencing of GMAP210.

These results demonstrate that GMAP210 is involved in the recruitment of the vesicular pool of LAT to the immune synapse but that it does not participate in the recruitment of CD3-ζ.

### GMAP210 controls signalosome formation and T-cell function

Others and us have shown that the recruitment of the vesicular pool of LAT is involved in T-cell activation^13–16,24^. We reasoned that GMAP210 by altering LAT recruitment to the IS should also control the activation of T lymphocytes. We activated GMAP210-silenced T cells with activating magnetic beads and purified the membranes associated with the beads as before (Fig. 2g) to study the composition of the signalosome. T cells transfected with control shRNA showed enrichment, in the signalosome, of LAT, PLCγ1, and SLP-76, as well as the vesicular SNARE VAMP7 (Fig. 4a). Of note, Western blot analysis of VAMP7 in the signalosome showed two bands. These two bands were observed in the signalosome shown in Fig. 2g and in the LAT-containing purified vesicles (Fig. 1c) but only one band was observed in the total cell lysates (“lysate” Fig. 1c, “input” Figs. 2g and 4a). This might reflect the enrichment in LAT-containing vesicles and in the signalosome of a pool of VAMP7 presenting post-translational modifications that modify its apparent molecular weight. In GMAP210-silenced cells the signaling complexes were incomplete. They contained less LAT, and SLP76 and almost no PLCγ1 (Fig. 4a and quantified in Fig. 4b) demonstrating the role played by GMAP210 in the formation of this signalosome. Moreover, they contain less VAMP7 suggesting a defect in the recruitment of VAMP7-bearing vesicles. In contrast, GMAP210 silencing did not alter the presence of CD3-ζ in the signalosomes (Fig. 4a), confirming the TIRF and confocal microscopy results, which showed normal recruitment of CD3-ζ to the synapse (Fig. 3). Activation of helper T lymphocytes by the TCR is characterized by the production of cytokines. To test if GMAP210 is indeed involved in T cell activation, we activated Jurkat T cells with APC or human CD4^+^ T lymphoblasts with different concentrations of anti-CD3 Abs and measured the production of cytokines (IL-2 for Jurkat and IFN-γ for primary T cells). Silencing of GMAP210 decreased the production of cytokines induced by TCR triggering at the protein (Fig. 4c: Jurkat and Fig. 4e: primary T cells) and mRNA level (Fig. 4d: Jurkat T cells). In contrast, GMAP210 silencing did not affect cytokine production induced by the PMA plus ionomycin (Fig. 4f, primary T cells) combination of pharmaceutical agents known to bypass LAT signaling^40^. These last results show that decrease of TCR-induced cytokine secretion by GMAP210 silencing is not due to a general effect on cytokine secretion but rather due to a defect in early TCR signaling. Altogether, these results show that GMAP210 is required for the formation of a functional TCR-induced signalosome and for T-cell function.

### GMAP210 captures vesicles carrying the VAMP7 vesicular SNARE

**Fig. 5:**
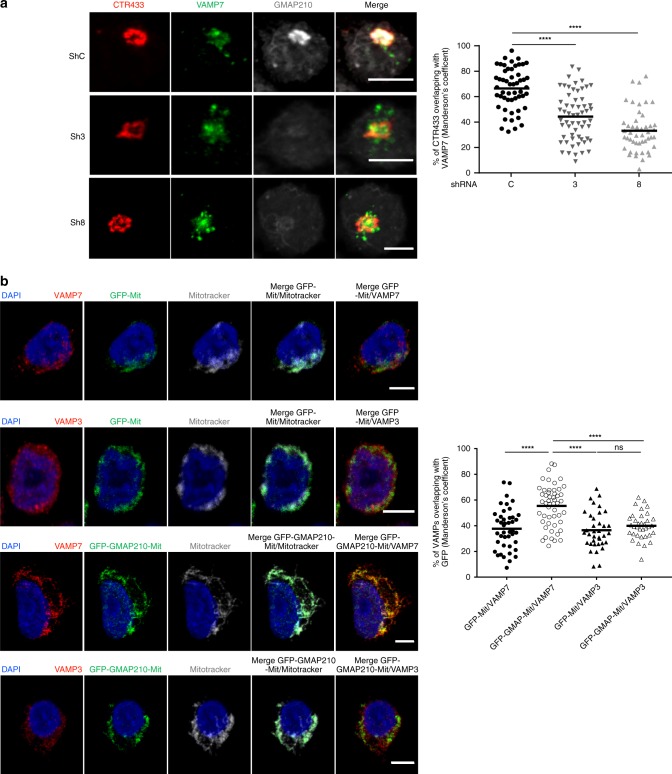
GMAP210 captures vesicles carrying VAMP7. **a** Confocal images showing the relative localization of VAMP7 (green) and CTR433 (red) in Jurkat cells expressing control (ShC) or GMAP210 specific shRNA (Sh3, Sh8) (GMAP210, nucleus in gray). Dot plot on the right side of the panel show the quantification of the percentage of CTR433 (Golgi marker) overlapping with VAMP7 (Manderson coefficient). Scale bar 5 μm. Each dot = one cell; horizontal lines = median. ****P < 0.0001 (one-way ANOVA). **b** Confocal images showing the localization of VAMP7 or VAMP3 (red) in Jurkat cells expressing a GFP-GMAP210-ActA chimera (GFP-GMAP210-Mit) or a GFP-ActA chimera (GFP-Mit), treated for 4 h with nocodazol (5 μg/mL) (nucleus in blue and mitochondria in Gray). Dot plot on the right side of the panel shows the quantification of the percentage of VAMPs (VAMP7 or VAMP3) overlapping with the GFP staining (GFP-mit or GFP-GMAP210-mit, Manderson coefficient). Scale bar 5 μm. Each dot = one cell; horizontal lines = median. ****P < 0.0001, ns: non-significant (one-way ANOVA). Data and Images represent two independent experiments in **a** and **b**

We have previously shown that the vesicular SNARE VAMP7 was required for the recruitment of LAT-containing vesicles to TCR-activation sites^13^. Moreover, results reported herein showed that recruitment of VAMP7 to the signalosome was decreased when GMAP210 was silenced (Fig. 4a, quantified in Fig. 4b). These results suggested that GMAP210 might bind VAMP7-bearing vesicles. We first observed that the distribution of VAMP7 in the Golgi was altered by GMAP210 silencing (Fig. 5a). The expression of VAMP7 was not altered in these cells (Supplementary Fig. 5a). Of note, the volume of the Golgi was not significantly modified suggesting that GMAP210 silencing did not grossly perturb the Golgi apparatus (Supplementary Fig. 5b). To directly test our hypothesis we then used a strategy, already described by others^28,48^, which consists in attaching GMAP210 to mitochondria and following the ectopic capture of different cargoes on mitochondria. Jurkat cells were transfected with a construct encoding a GFP chimeric GMAP210 molecule tagged with the C-terminal hydrophobic anchor of ActA, which anchors GMAP210 to mitochondria, or with a construct encoding GFP tagged the same way as control (GFP-Mit)^48^. Transfected Jurkat cells were treated with nocodazole, because previous studies showed that capture of vesicles by golgins on mitochondria was more efficient when microtubules were depolymerized^28,49^. The ectopic localization of GMAP210 to mitochondria induced the displacement of VAMP7 to the mitochondria (Fig. 5b). This was specific of VAMP7, since no displacement of VAMP3, another vesicular SNARE that controls TCR^17^ but not LAT recruitment^13^ to the immune synapse (Fig. 5b), was observed. Unfortunately, we could not realize this test in activating conditions to see if TCR activation increased the binding of GMAP210 to VAMP7-decorated vesicles. Indeed, this assay requires depolymerization of microtubules, which alters T-cell activation and LAT transport^50^. These results strongly support that GMAP210 specifically binds VAMP7 “decorated” vesicles.

### GMAP210 tethering activity controls vesicular traffic of LAT

**Fig. 6:**
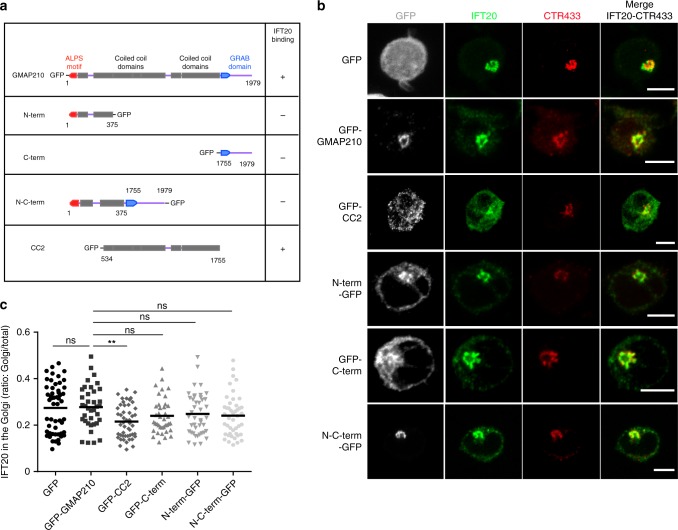
IFT20 localization in the Golgi in Jurkat cells expressing different GMAP210-GFP domains. **a** Schematic representation of GMAP210 and fusion constructs with different IFT20-binding capacity. **b** Confocal images showing the relative localization of IFT20 (green) and CTR433 (red) in Jurkat cells expressing GFP, GFP-GMAP210, or different GMAP210 domains coupled to GFP. **c** Quantification of the ratio of IFT20 fluorescence present in the Golgi versus total fluorescence in the different conditions. Scale bar 5 μm. Each dot = one cell; horizontal lines = median. **P < 0.01, ns: non-significant (one-way ANOVA). Data and images represent two independent experiments in **b** and **c**

GMAP210 binds the intraflagellar protein IFT20^35,36^ and anchors IFT20 to the Golgi complex^35^. We previously showed that IFT20 regulates TCR recycling and LAT recruitment to the immune synapse^51,52^. We thus investigated if GMAP210 plays a role in the localization of IFT20 in T lymphocytes. As already observed in ciliated cells^35^, absence of GMAP210 induced a dispersion of IFT20 from the Golgi (Supplementary Fig. 6a and quantified in Ssupplementary Fig. 6b). This dispersion of IFT20 was also observed in cells overexpressing the IFT20-binding CC2 domain (amino acids 534–1779) coupled to GFP^31^ (Fig. 6), suggesting that, in T lymphocytes, GMAP210 retains IFT20 close/at the Golgi via its CC2 domain. Anchored to the Golgi membranes by its C-terminal domain^30,32,53^, GMAP210 which binds vesicles through its N-terminus domain^33,34,49,54^ tethers them to the Golgi. To investigate the role of this tethering activity, we overexpressed the N-terminal domain encompassing amino acids 1–375, the C-terminal domain (amino acids 1778–1979) and a shorter version of GMAP210 that contains both N-term and C-term domains but lacks most of the coiled-coil domain (Fig. 6a). As described^31^, all these constructs were localized to the Golgi (Fig. 6b). In contrast to the overexpression of CC2, none of them displaced IFT20 from the Golgi (Fig. 6b, c).

**Fig. 7:**
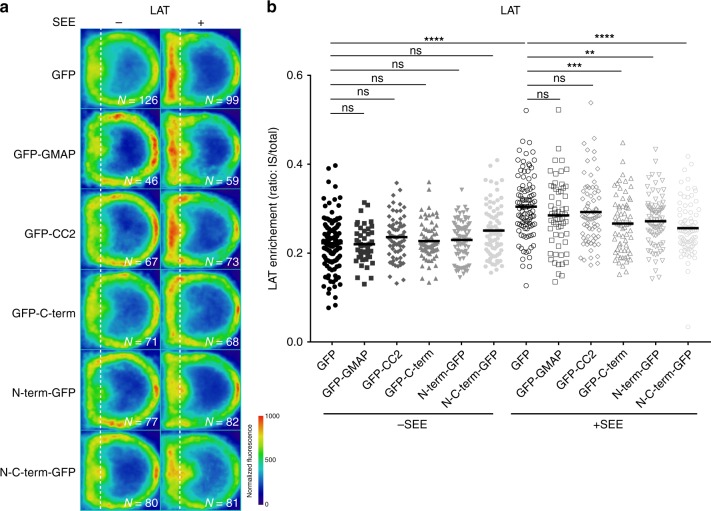
GMAP210 tethering activity controls LAT recruitment at the immune synapse. **a**, **b** Confocal images (left panels) and quantification (right panels) of the enrichment of LAT (**a**, **b**) at the immune synapse (depicted by the dotted white line) in Jurkat “mean cells” expressing GFP alone, GMAP210-GFP, or different GMAP210 domains coupled to GFP and interacting for 30 min with Raji cells left unpulsed (−, unactivated state) or pulsed with SEE (+, immune synapse formation). *N* = number of cells constituting the mean image. Horizontal line represents median. **P < 0.01, ***P < 0.001, ****P < 0.0001, ns: non-significant (one-way ANOVA). Data are representative from two independent experiments

We then studied the effect of the overexpression of the different GMAP210 constructs on LAT recruitment and phosphorylation at the immune synapse. Although CC2 overexpression induced a dispersion of IFT20 from the Golgi (Fig. 6), it did neither alter LAT recruitment (Fig. 7a, quantification in Fig. 7b) nor LAT phosphorylation (Supplementary Fig. 7a, quantified in Supplementary Fig. 7b) to the immune synapse. These results suggest that although GMAP210 is involved in the localization of IFT20 to the Golgi apparatus, its binding activity is not required for LAT trafficking.

In contrast, overexpression of the N-terminal and C-terminal domains of GMAP210, as well as the short version of GMAP210 induced a significant decrease in LAT recruitment to the immune synapse (Fig. 7a, quantification in Fig. 7b). This was accompanied by less phosphorylated form of LAT at the immune synapse (Supplementary Fig. 7a, quantified in Supplementary Fig. 7b). In contrast overexpression of the short version of GMAP210, which inhibits LAT recruitment and phosphorylation at the immune synapse, did not affect the phosphorylation of ZAP70 at the immune synapse (Supplementary Fig. 7c and quantified in Supplementary Fig. 7d). Altogether, these results suggest that the tethering activity of GMAP210 is involved in the delivery of vesicular LAT to the immune synapse.

### Traffic of ectopically expressed LAT to the primary cilium

**Fig. 8:**
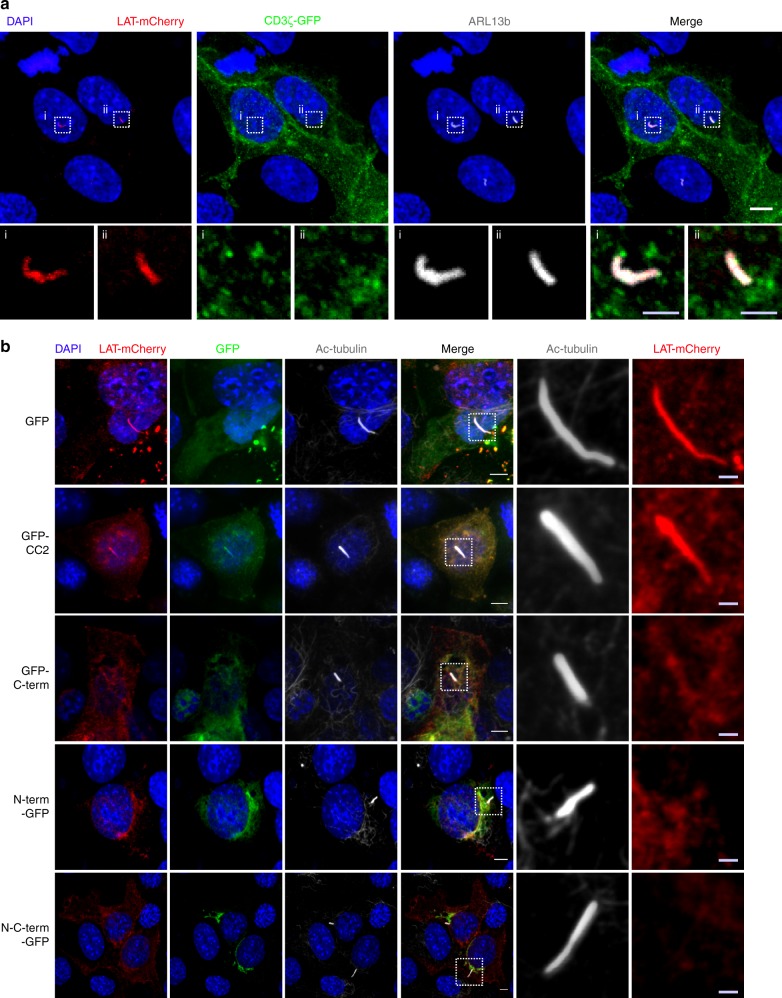
Specific recruitment of LAT to the primary cilium depends on GMAP210 tethering activity. **a** Confocal images showing the localization of ARL13b (gray) and LAT-mCherry (red) or CD3ζ-GFP (green) in ciliated mIMCD-3 epithelial cells. Staining of the nucleus by DAPI in blue. White squares indicate the magnified regions presented underneath that shows the primary cilia. White scale bars: 5 μm, gray scale bars: 1.5 μm. **b** Confocal images showing the relative localization of LAT-mCherry (red) and acetylated-tubulin (gray), in mIMCD-3 cells co-expressing GFP alone, GMAP210-GFP, or different domains coupled to GFP. White squares indicate the magnified regions presented on the right that shows the primary cilia. White scale bars: 5 μm, gray scale bars: 1.5 μm. Images representative of two independent preparations in **a** and one preparation in **b**

GMAP210 was also shown to control trafficking of specific cargos to the primary cilium^35,37,38^. We reasoned that LAT, which is not expressed in ciliated cells, might follow a transport pathway, which is used by cargoes specifically going to the cilium. To test this hypothesis, we expressed LAT in the mIMCD-3-ciliated cells. LAT was transported to the cilium where it co-localized with ARL13B, a marker of the cilium^55^, demonstrating that the intraciliary trafficking machinery could take care of the vesicular transport of LAT (Fig. 8a). This was rather specific since once introduced in ciliated cells, the transmembrane protein CD3-ζ, whose recruitment to the immune synapse does not depend on GMAP210 (Fig. 3) and VAMP7^13^, was not transported to the cilium (Fig. 8a). Overexpression of the GMAP210-encoding constructs described earlier also showed that the tethering activity of GMAP210 was involved in transport of LAT to the cilium (Fig. 8b). As observed for the synapse, overexpression of the CC2 domain that binds IFT20 did not block LAT trafficking to the cilium.

These results show that, when ectopically expressed in ciliated cells, LAT traffics to the primary cilium. Moreover, they show that the tethering activity of GMAP210 to the Golgi controls LAT delivery to the primary cilium highlighting the similarities between transport to the immune synapse and to the cilium.

## Discussion

We show herein that GMAP210 by tethering vesicles containing LAT to the Golgi helps their correct delivery to the immune synapse. By doing so it organizes the formation of LAT-containing signalosomes involved in T lymphocyte activation revealing a new molecular player in the formation of the immune synapse.

Several studies have investigated the cellular function of GMAP210. In some cells, depletion of GMAP210 has been reported to cause Golgi fragmentation without defect in secretory trafficking^53,56^. This is not the case in human T cells, in which no defect in the Golgi morphology and volume are observed (Supplementary Fig. 5b). GMAP210 is a long coiled-coil molecule that binds to the cis-Golgi via its C-terminal domain^30–32^ and captures vesicles through its N-terminal domain^34,49,54^. Using the mitochondrial relocation assay, it was shown to capture vesicles containing Golgi, as well as endoplasmic resident proteins^28^. We show herein that it also captures vesicles bearing VAMP7 (Fig. 5b), which is expressed on Golgi membrane in T cells (Fig. 5a). Concerning the specificity of vesicular binding of GMAP210, GMAP210 contains an ALPS motif at its N-terminal end. This motif does not present a sequence-specific interaction site, but rather senses the curvature of the vesicles, preferentially binding highly curved liposomes (radius < 50 nm) with monounsaturated lipids^34^. The N-terminal domain of GMAP210 also contains a sequence-specific interaction, suggesting that this golgin can bind two different types of vesicles^54^. It is worth noting that the sizes of the vesicles containing both LAT and GMAP210, found in our study (Fig. 1b, d), are compatible with the size preference of the GMAP210 ALPS domain. This gives us precious insights and hypothesis to be tested on the potential lipid composition of the LAT-containing vesicles, the cargoes they may contain and the consequences this composition may have on the formation of the immune synapse.

**Fig. 9:**
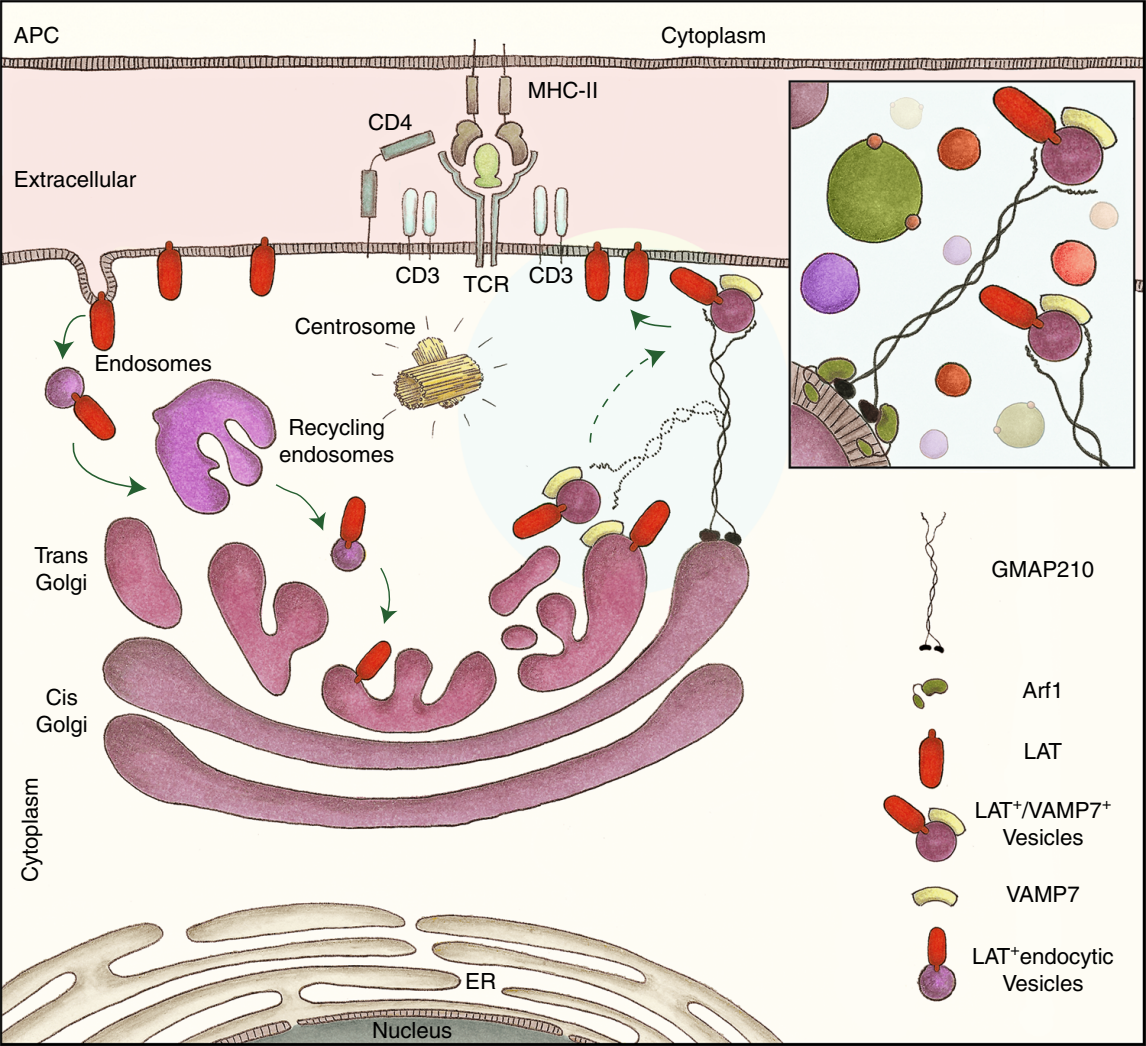
Graphical abstract: GMAP210 facilitates the delivery of vesicles containing LAT to the immune synapse. Upon TCR triggering, LAT is internalized in recycling endosomes. This endocytic pool of LAT is “retrotransported” to the Golgi apparatus^24^, where it meets the vesicular SNARE VAMP7 that is involved in LAT trafficking^13^. GMAP210, which binds the Golgi through Arf1, sorts and captures the LAT/VAMP7 vesicles via its N-terminal domain (inset). The long coiled-coil domain of GMAP210 then brings LAT-containing vesicles close to the immune synapse

Humans with mutations in GMAP210 and GMAP210-knockout mice die early on from a severe skeletal dysplasia^57^. This is associated with a defective trafficking of some cargo proteins in the early secretory pathway of chondrocytes^57,58^. The role of GMAP210 in the early secretory pathway, i.e. both anterograde and retrograde trafficking has been confirmed in other cell types^59,60^. We recently showed that the endocytic LAT is following a retrograde pathway back to the Golgi apparatus. This transport pathway which exists at steady state is increased upon TCR activation and is crucial for LAT transport to the immune synapse^24^. These results associated to the data reported herein suggest the following model (Fig. 9): Upon TCR activation, LAT is endocytosed and transported through the retrograde transport pathway to the Golgi. From there, vesicles-containing LAT and VAMP7 are budding and are captured by GMAP210 to be delivered back to the immune synapse. This happens 10–15 min after activation and corresponds to an active phase of vesicle recruitment^45^. The Golgi apparatus is polarized close to the immune synapse, as reported early on^61^ and showed by electron microscopy^43,44,62^. Yet, it does not dock to the immune synapse, as shown by the absence of GM130, a marker of the Golgi, in the evanescent field of the TIRFM. Thus the long coiled-coil domain of GMAP210, 200–300 nm^31^, could allow the proximity and docking at the immune synapse of the LAT/VAMP7-vesicles bound to the N-terminal domain of GMAP210. This pool of vesicular LAT delivered in a GMAP210-dependent manner participates to the formation of LAT signalosomes (Fig. 4a, b).

GMAP210 has also been shown to bind IFT20 in several cell types including T lymphocytes^35,36^ and to anchor this protein to the Golgi^35^. This intraflagellar transport (IFT) protein, which regulates the assembly of the primary cilium, also regulates trafficking of the TCR^51^ and LAT^52^ to the immune synapse. Yet, our results suggest that the IFT20-binding activity of GMAP210 is not involved in the vesicular transport of LAT (Fig. 7). Moreover, whereas IFT20 controls TCR/CD3-ζ recruitment to the immune synapse^51^, GMAP210 does not (shown here Fig. 3 and Supplementary Fig. 3 and in ref. ^36^). Thus, at least some effects of IFT20 on the formation of the immune synapse formation do not rely on its binding to GMAP210.

One interesting aspects of our study is that, as reported for transport to the cilium^37^, GMAP210 does show some specificities for vesicles and cargoes delivered to the immune synapse. Indeed, GMAP210 silencing does not affect CD3-ζ recruitment to the immune synapse (Fig. 3), this might be due to the difference in the “nature” of the vesicles containing CD3-ζ.

We now have to characterize if GMAP210 controls the delivery of other molecules to the immune synapse but also if other golgins are involved in immune synapse formation.

Although, T lymphocytes are non-ciliated cells, morphological similarities have been shown between the immune synapse and the primary cilium^63,64^. In particular, the central role of controlled vesicular trafficking in signaling by these structures has been demonstrated^65–67^. Our results further extend this analogy. Indeed, we show that LAT, an emblematic signaling molecule of the immune synapse, is transported to the primary cilium (Fig. 8a) when ectopically expressed in ciliated cells. Like for the immune synapse, LAT trafficking to the cilium depends on the tethering activity of GMAP210 (Fig. 8b). Hence, studying LAT trafficking to the immune synapse can bring information on vesicular trafficking to the cilium.

Together, our results reveal a new player and a new mechanism in transport to-and formation of-the immune synapse that may also be a key player of cilium generation. These results open a new field of research that may have valuable implications in the study of ciliopathies and immunodeficiencies.

## Methods

### Cells

Jurkat T cells (validated by SSTR method present 88% of homology with DSMZ Leibniz ACC 282), JCAM2.5 cells stably expressing the mouse LAT-*Strep*tag construct^25^, Jurkat expressing the CD3-ζ-GFP chimera described elsewhere^68^, and Raji B (ATCC, CCL-86) cells were cultured at 37 °C 5% CO_2_ in RPMI 1640 Glutamax (Gibco, 61870-010) supplemented with 10% fetal calf serum (FCS, Lonza, DE14-801F, lot no. 0SB017) and were passed every 2–3 days at ~0.5 × 10^6^ cells/mL.

Inner medullar collecting duct cells (IMCD3, ATCC CRL-2123), a kind gift from Alexandre Benmerah (Laboratory of Hereditary Kidney Diseases, Imagine Institute, Paris, France), were grown in Dulbecco’s modified Eagle’s medium DMEM-F12 1:1 (GIBCO, 31331-028) supplemented with 10% FCS for basic cell culture conditions.

Mononuclear cells were isolated from peripheral blood of healthy donors on a ficoll density gradient. Buffy coats from healthy donors (both male and female donors) were obtained from Etablissement Français du Sang (Paris, France) in accordance with INSERM ethical guidelines. Human total CD4+ isolation kit (Miltenyi Biotech, 130-096-533) was used for the purification of T cells. To obtain lymphoblastoid effector T cells^13^, six-well plastic plates were coated overnight at 4 °C with αCD3 (OKT3 clone, eBioscience, 16-0037-85, 2.5 μg/mL final concentration in 1.3 mL). Wells were washed and 5.4 × 10^6^ purified primary human total CD4+ T cells were then plated per well in the presence of soluble anti-CD28 (LEAF Purified anti-human CD28 from CD28.2 clone, Biolegend, BLE302923) at 2.5 μg/mL final concentration and recombinant human IL-2 (20 U/mL, Novartis, Basel, Switzerland) in RPMI culture medium supplemented with 10% FCS, penicillin/streptomycin (100 U/mL, Gibco, 15-140-122), 10 mM HEPES (Gibco, 15630-080) and 0.05 mM β-mercaptoethanol, (Gibco, 31350-010) at 37 °C, 5% CO_2_.

### Reagents and antibodies

Recombinant Staphyloccocus Enterotoxin type E (SEE, Cellgenetech, MBS1112600), Ionomycin (407950; Calbiochem), PMA (Sigma-Aldrich, 79346) and Poly-l-lysine (Poly-Lys, Sigma-Aldrich, P8920) were used.

For detailed information on dilutions, companies, and reference numbers see Supplementary Table 1.

### Production of lentiviruses and infection of CD4^+^ T cells

Non-replicative VSV-g pseudotyped lentiviral particles were produced by transfecting HEK-293T cells with Gag, Pol, rev, encoding plasmid (pPAX2), envelop encoding plasmid (pMD2.G) and either the HA-Tev-LAT construct^13^ encoded in a pWXLD vector, or short hairpin RNA (shRNA) sequences encoded in pLKO.1 plasmid: Non-targeting control shRNA (shC, Sigma-Aldrich, Mission shRNA SHC002), GMAP-210-specific shRNA, sh3 (Sigma-Aldrich, Mission shRNA, sequence GCAAAGGAACAAGAACTCAAT), and sh8 (Sigma-Aldrich, Mission shRNA, sequence GCAGAAGATAGAGAGGCTAAACT). Lentivirus were recovered in supernatant after 2 days and concentrated. 5 × 10^6^ Jurkat T cells were infected for 24 h, cells infected with shRNA encoding virus were selected in puromycine (2 µg/mL, InvivoGen, ant-pr) and used 5 days post infection.

Primary human CD4+ T cells were activated in six-well plates coated with anti-CD3 (2.5 μg/mL) in the presence of soluble anti-CD28 (2.5 μg/mL) and recombinant IL-2 (20 U/mL). Concentrated virus was added 36–48 h later. Cells were washed and then put in fresh medium with IL-2 (20 U/mL) and puromycin (2.5 μg/mL) 24 h later and used 72 h later.

### Plasmids

The plasmid encoding LAT-mCherry and CD3ζ-GFP were reported previously^13,68^. Plasmids encoding chimeric molecules GFP-GMAP210, N-term-GFP, GFP-C-term, N-C-term-GFP, GFP-CC2, GFP-GMAP-Mit, GFP-Mit are described elsewhere^31,48^.

### Transfection

Jurkat T cells were transfected using the Amaxa Cell Line Nucleoefector Kit V (Lonza, VCA-1003). To do so, 5 × 10^6^ cells were washed, resuspended in 100 µL of nucleoefector solution and combined with 5–10 µg of DNA. Cells were passed into the electroporation cuvettes and then electroporated (Amaxa program X-005). Cells were then incubated at room temperature for 10 min, recovered and diluted in warmed RPMI supplemented with 10% FCS and cultured for 24 h at 37 °C, 5% CO_2_.

To transfect IMCD3, cells were grown to confluence on 12 mm coverslips in 24-wells plate. Medium was removed and 500 µL of Optimem (GIBCO, 31985-047) was added. In a final volume of 50 µL of Optimem, 2 µL of Lipofectamine 2000 (Invitrogen, 11668019), and 2 µg of DNA were mixed and incubated for 10 min at room temperature. The mix Lipofectamine/DNA was added and cells were incubated for 24 h to induce ciliogenesis.

### Preparation of lysates from Jurkat or Human CD4+ T lymphoblasts

1 × 10^6^ cells/mL of Jurkat T-cells or human lymphoblasts were washed three times with cold PBS and incubated on ice for 20 min in 30 µL ice-cold lysis buffer (50 mM Tris pH 8, 150 mM NaCl, 1,5 mM MgCl_2_, 1% Glycerol, 1% TritonX100, 0.5 mM EDTA pH 8, 5 mM NaF) supplemented with a protease inhibitor cocktail (Sigma-Aldrich, 11873580001). Post-nuclear lysates were obtained by centrifugation at maximum velocity for 15 min at 4 °C. Laemmli Sample Buffer (BIORAD, 161-0747) and reducing agent (Thermo Fisher Scientific, NP0009) were added and samples were heated at 95 °C for 5 min and kept at −20 °C before immunoblot analysis.

### Preparation of LAT-containing membranes

JCAM2.5 LAT-deficient Jurkat cells (150 × 10^6^) expressing the mouse LAT-StrepTag (LAT-TST) protein^25^ or non-transfected JCAM.2.5 were washed, resuspended in RPMI at 100 × 10^6^/mL and activated with soluble anti-CD3 (125 ng/mL) and anti-CD28 (250 ng/mL) antibodies for different times (0, 5, 15, and 30 min). The activation was stopped by adding cold PBS and the cells were centrifuged at 1800 × *g* at 4 °C for 5 min. The cell pellet was then suspended in homogenization buffer (0.25 M sucrose, 10 mM Tris–HCl pH 7.4, 1 mM EDTA) supplemented with a EDTA-free protease inhibitor cocktail (Roche, 1123000) and a phosphatase inhibitor cocktail (Thermo Scientific, 78420). Cell breakage was induced on ice by 25 successive stokes of a Dounce homogenizer. The cell suspension was then passed 15 times through a 25GA needle to achieve cell disruption and centrifuged for 5 min at 900 × *g* at 4 °C to remove nuclei and unbroken cells. The supernatant was transferred into Ultra-clear centrifugation tubes (Beckman Coulter) and centrifuged at 65,000 × *g* for 1 H at 4 °C in a SW55Ti rotor (Beckman Coulter). The pellet was suspended in 1.2 mL of homogenization buffer supplemented as before and passed several times through a 25GA needle to ensure complete resuspension of the membranes. This suspension was transferred into a new tube and mixed with 1.2 mL of a 60% solution of Optiprep/iodixanol (Axis-shield) to reach a 30% iodixanol suspension. The Optiprep solution was diluted extemporaneously into 0.25 M sucrose, 60 mM Tris–HCl pH 7.4, 6 mM EDTA to prepare 1.3 mL of a 20% solution and 1.2 mL of a 10% solution. The 20% and the 10% iodixanol solutions were layered successively on top of the 30% suspension and centrifuged at 350,000 × *g* for 3 h at 4 °C in a SW55Ti rotor without brake when stopping. Ten fractions of 490 µl were collected from the top of the tube. To purify LAT-*TST*-associated membranes, the third fraction (fraction 3) was incubated for 90 min at 4 °C on a rotating wheel with pre-washed *Strep*-Tactin Sepharose resin in presence of protease and phosphatase inhibitors. Resin was washed in *Strep*Tag washing buffers (Buffer W: Tris–HCl 100 mM, NaCl 150 mM, EDTA 1 mM, pH 8.0) and suspended in RIPA lysis buffer before being submitted to SDS–PAGE and immunoblot analysis.

### Purification of LAT-signalosome

Jurkat cells (5 × 10^6^) were resuspended in 200 µl of RPMI medium, and magnetic beads (1 × 10^7^) coated with anti-CD3 and anti-CD28 (Gibco, 11132D) were added in a volume of 100 µl. Beads were incubated with T cells for the appropriate time at 37 °C. Activation was stopped with the addition of 500 µl cold PBS, and 80 µl (1/10) of samples were collected as ‘input’ and lysed as described above (‘'Preparation of lysates from Jurkat or human CD4^+^ T lymphoblasts' section). Bead-cell conjugates were then magnetically restrained, resuspended in 500 µl of ‘freeze–thaw’ buffer (600 mM KCl, 20 mM Tris, pH 7.4, and 20% glycerol) supplemented with, EDTA-free Protease Inhibitor Cocktail Tablet (Roche, 1123000). Samples were submitted to seven cycles of freezing and thawing. After the final cycle, 5 µl benzonase (Novagen, 2733353) was added, followed by incubation for 20 min at room temperature. Samples were magnetically restrained to purify the bead-attached proteins and then were washed five times in the supplemented ‘freeze–thaw’ buffer described above. Bead-associated proteins were resuspended in lysis buffer and separated by SDS–PAGE and analyzed by immunoblot.

### Immunoblot analysis

Samples were resolved on NuPage 4–12% Bis–Tris gel (Thermo Fisher Scientific, NP0323BOX) and liquid transferred (Thermo Fisher Scientific, NP00061) on PVDF membranes (Bio-Rad, 162-0177). After blocking with TBS 0.05% Tween20 5% BSA for 1 h 30 min on rocking platform shaker, membranes were incubated overnight at 4 °C with primary antibodies. Membranes were washed three times with TBS 0.05% Tween and incubated for 40 min in TBS 0.05% Tween on rocking platform shaker with the secondary antibody. Bound antibodies were revealed using the Clarity^TM^ Western ECL substrate (Bio-Rad, #170-5061), according to the manufacturers’ directions. The intensity of the bands was quantified by densitometry using Image Lab 5.2.1 software (Bio-Rad Laboratories) and was expressed as arbitrary units. All original gel images are included in the Source Data file.

### Coverslips and dishes preparation for immunofluorescence assay

12mm ø coverslips (VWR, 631-0666) for fixed cells or fluorodishes (World Precision Instrument Inc., FD35-100) for live imaging were pre-coated with 0.02% of poly-l-Lysine for 20 min at room temperature and were washed three times in water before being dried and kept for maximum 2 days.

### Preparation of Jurkat T cells and Raji B cells conjugates

Raji B cells were washed, resuspended at a concentration of 1 × 10^6^ cells/mL in RPMI without FCS and labeled with CellTracker™ Blue CMAC dye (10 µM, Thermo Fisher, C2110) for 20 min at 37 °C. Labeling was stopped with RPMI 10% FCS and cells were washed once and resuspended at 1 × 10^6^ cells/mL. Cells were pulsed with SEE (100 ng/mL) or left untreated for 30 min at 37 °C before being washed once and resuspended at a concentration of 1 × 10^6^ cells/mL. 100,000 Raji cells were incubated on coverslips for 30 min, washed once with warmed PBS and 150,000 Jurkat cells resuspended in RPMI 10% FCS were added for 30 min. Coverslips were washed once with cold PBS before fixation.

### Mitochondrial capture assay in cells expressing GFP-GMAP-Mit

Jurkat cells were washed, resuspended at 1 × 10^6^ cells/mL and incubated 4 h with nocodazol (5 μg/mL) in RPMI containing 10% of FCS at 37 °C. 150,000 Jurkat cells were then incubated on coverslip for 30 min, washed once with cold PBS and fixed.

### Fixed and live TIRF microscopy

Poly-l-Lysine-coated coverslips were left untreated or coated overnight at 4 °C with αCD3ε αCD28, washed three times and pre-warmed at 37 °C for 10–15 min. 150,000 Jurkat or primary CD4+ T cells were incubated on coated coverslips for 15 min at 37 °C before being washed once with cold PBS and fixed. For live imaging, fluorodishes were coated following this same protocol. 200,000 Jurkat T cells were plated and images for 491 and 561 channels were acquired every 3 s.

### Fixation

Cells were fixed with 4% paraformaldehyde (Life technologies, FB002) for 15 min at room temperature, washed once in PBS and excess of paraformaldehyde was quenched for 10 min with PBS 10 mM Glycine (Thermo Fisher Scientific, G8898). Coverslips were kept at 4 °C in PBS until permeabilization and staining.

### Staining and mounting

Cells were permeabilized for 30 min at room temperature with PBS 0.2% Bovine Serum Albumin (BSA, Euromedex, 04-100-812) 0.05% Saponin (Sigma-Aldrich, S4521). Cells were then incubated for 1 h at room temperature with primary antibody, followed by washing three times with PBS 0.2% BSA 0.05% Saponin and incubated protected from light for 20 min in the same buffer with spinned secondary antibodies. After washing once with PBS BSA Saponin, and once with PBS, coverslips were soaked three times in PBS, three times in water, and mounted on slides.

For regular confocal microscopy, coverslips were mounted with 4–6 µL of Fluoromount G (SouthernBiotech, 0100-01) on slides (KNITTEL Starfrost) and dried overnight protected from light before microscope acquisition.

For TIRF microscopy, following staining, with secondary antibody, coverslips were soaked in PBS and mounted with 4–6 µL of PBS, sealed with uncolored nail polish and dried for 15 min before acquisition.

### Microscopes and Images analysis

Confocal microscopy was achieved using a Laser Scanning Confocal microscope (LSM780, Zeiss) from the PICT-IBiSA @Pasteur Imaging facility at Institut Curie, equipped with 40X or 100X Plan Apo objectives (numerical apertures, 1.35) and a 1-airy unit pinhole size was used. Single plane images or Z-stack of images were acquired (pixel size around 70 nm). TIRF microscopy was performed using an inverted Nikon microscope Ti-E from the Nikon Imaging Center at Institut Curie-CNRS equipped with a 100X CFI Apo TIRF objective (numerical aperture of 1.49), 491, 561, and 642 nm lasers, and an EMCCD 512 Evolve camera (Photometrics, pixel size 0.16 µm). For live experiment, temperature was constantly sustained at 37 °C and one image was acquired every 3 s. Images were analyzed on Fiji and ImageJ software and compatible scripts were generated for automated or semi-automated analysis.

### Recruitment at the immune synapse and “Mean Cell” creation

Single images corresponding to the middle planes of conjugates were extracted from Z-stack. T cells were cropped and oriented in the same way regarding their synapse (script#1). Obtained T cell images were grouped by condition (shRNA ± SEE) and fluorescence intensities were normalized by the mean fluorescence intensity (MFI). Images were then resized to the smallest image size in order to create a normalized stack of images for each group (script#2). All groups were normalized (size and intensity) before being compared. Stacks of aligned cells were finally projected (averaging method) giving single plane “mean cells” (script#3). Stacks were resized to obtain a 1-pixel height stack by averaging the fluorescence intensity of the total height of each image. Projections of the 1-pixel resized stacks were obtained based on average and standard deviation methods and pixel intensities profiles were performed along projections width (script#4). In order to get a cell-by-cell quantification, we also computed an enrichment ratio at the synapse. This enrichment was defined as the ratio between the total cell fluorescence and the fluorescence in the synaptic region (rectangle at the synapse representing 20% of the total cell). (script#3).

### Enrichment of P-LAT and P-ZAP70 in Jurkat-Raji conjugates

P-LAT and P-ZAP70 enrichment at the T cell–APC contact site was quantified as described previously^13^. Briefly, in each cell, the MFI at the IS of P-LAT or P-ZAP70 was divided by the average of the mean intensities measured in three regions of the same size at the plasma membrane outside of the IS (IS-to-membrane ratio).

### Recruitment of molecules to the immune synapse by TIRFM

Before imaging cells, TIRFM angle was set up to provide an evanescent field of fixed thickness (<120–150 nm). Illuminated puncta were imaged, and the background was subtracted (50 pixels, rolling ball radius) for each acquired image. Cells were manually segmented to obtain regions of interest (ROIs) and their areas were measured. Then, within each ROI, puncta present in the evanescent field were defined as signal intensity maxima detected by using the “Find Maxima…” function, for which a value of noise tolerance was arbitrarily set according to background from experiment to experiment (values around 5000 in most experiments). Using this method allowed the discrimination of maximas coming from clusters (local bright patches at the plasma membrane or just below in the limit of thickness of the evanescent field) from a homogeneous signal. The number of “maximas” was then counted for each ROI, giving a cell-by-cell quantification of the number of puncta or density of puncta at or below the plasma membrane.

### Colocalization assay on TIRFM images by Pearson coefficient

Illuminated puncta were imaged, and the background was subtracted (50 pixels, rolling ball radius) for each acquired image. Cells were manually segmented to obtain regions of interest (ROIs). Within each ROI, colocalization assays were performed using the JACoP plugin for ImageJ64 to obtain Pearson coefficient.

### Analysis of LAT-GMAP210 colocalization in conjugates

Z-stack (0.4 µm) images of similarly dimensioned conjugates were chosen. In that z-stack, a ROI surrounding the GMAP210 staining was defined. Within each ROI, colocalization assays were performed using the JACoP plugin for ImageJ64 to obtain Pearson coefficient.

### Analysis of Golgi volume in Jurkat cells

Z-stack (0.4 μm z-step) images of T cells were chosen. Then, ROI surrounding the Golgi was defined for each cell based on CTR433 staining. Within each ROI, masks based on CTR433 staining, were created by automatic thresholding. To finish, Golgi volume was measured using the “3D Objects Counter” plugin from Fiji.

### VAMP7-CTR433 colocalization in GMAP210-depleted cells

Z-stack (0.5 µm) images of similarly dimensioned Jurkat cells were chosen. In that z-stack, a ROI surrounding the Golgi was defined based on CTR433 staining. Within each ROI, masks based on both CTR433 and VAMP7 stainings were created by thresholding. Automatic colocalization assays were performed with Mander’s overlap coefficient, using the JACoP plugin for ImageJ64.

### Analysis of VAMPs capture in the mitochondria

Z-stack (0.5 µm) images of similarly dimensioned Jurkat cells were chosen. In that z-stack, cells were manually segmented to obtain a ROI. In each ROI, masks based on both GFP^10^ and VAMPs stainings were created by thresholding. Automatic colocalization assays were performed with Mander’s overlap coefficient, using the JACoP plugin for ImageJ64.

### Analysis of IFT20 displacement in Jurkat cells

Middle planes images from Z-stack images of Jurkat T cells were chosen. Cells were manually segmented. For each cell, a Golgi mask was defined using the CTR433 staining. To finish, IFT20 total fluorescence was measured inside the cell and inside the Golgi region. IFT20 displacement was defined as the ratio of Golgi-associated fluorescence vs. total-associated fluorescence.

### Electron microscopy

Rabbit anti-LAT used at 1:50 (Millipore, 06-807), rabbit anti-GMAP210 1:80 (Gift from Michel Bornens, Institute Curie, Paris, France), PAG 1:50 (protein A gold, Utrecht University, the Netherlands), F(ab)2 fragment goat anti-rabbit 6 nm 1:20 (Aurion). Immunoelectron microscopy on ultrathin cryosections was performed by the Tokuyasu method^69^. Sections were examined on a Tecnai Spirit electron microscope (FEI, Eindhoven, The Netherlands) equipped with a Quemesa camera (EMSIS GmbH, Münster, Germany). For immunolabeling on whole-mount vesicles, fraction 3 from suspension in PBS (from JCAM2.5 LAT-deficient Jurkat cells expressing the mouse LAT-*Strep*Tag) was deposited on formvar-carbon-coated cooper/palladium grids as described previously^70^.

### Interleukin-2 secretion and production assay in Jurkat cells

Jurkat T cells and Raji B cells were washed and resuspended at 1 × 10^6^cells/mL. 100 µL Jurkat cells and 50 µL of Raji cells were mixed in a 96-well plate, flat bottom (TPP, 92096). 50 µL of SEE at the final indicated concentrations were added for 6 h. Supernatants were recovered and tested for IL-2 by ELISA (BD OptEIA, 555190). Total mRNA was isolated from cells with NucleoSpin RNA kit (Macherey Nagel, 740-955) and IL-2 mRNA expression was assessed by quantitative Polymerase Chain Reaction using Taqman method and IL-2 mRNA targeting primers (Thermo Fisher Scientific, SM-IL2-Hs00174114_m1 for IL-2 and SM-RPL34-Hs00241560_m1 for the housekeeping).

### Interferon-γ secretion primary CD4+ T cells

Human CD4+ T lymphoblasts were subjected to gradient centrifugation to remove dead cells. 100,000 cells were activated either on 96-well plate, flat bottom (TPP, 92096) coated overnight at 4 °C with αCD3εαCD28 at the indicated concentration or with a soluble combination of 10 ng/mL of PMA and 1 µg/mL of ionomycin. Supernatants were recovered after 6 h and tested for IFN-γ by ELISA (BD OptEIA, 555142).

### Flow cytometry

Cells were centrifuged and transferred to conical bottom plate (Greiner Bio-One, 650101), stained for 20 min in cold PBS with Fixable Violet Dead Cell Stain Kit (1/4000, Invitrogen, L34955) and washed in FACS Buffer (PBS 0.5% BSA 2 mM EDTA). Extracellular staining was performed in FACS buffer for 30 min on ice. For surface marker expression of human cells, antibodies anti-CD28, anti-TCRα/β, anti-CD3, anti-CD4, and anti-HA were used and are described in Supplementary Table 1. After staining, cells were washed in FACS buffer and fixed with Cytofix/Cytoperm (BD Biosciences, 554714).

For intracellular cytometry, cells were washed twice in Perm/Wash buffer (BD, 554723), and incubated for 1 h in ice with anti-LAT or anti-CD45. In the next step, cells were washed twice with perm/wash followed by staining with the correspondent secondary antibody (see Supplementary Table 1).

Finally, cells and compensation beads (eBioscience, 01-1111-42) were acquired with BD FACS Verse and MACS Quant (Miltenyi) flow cytometer and data were analyzed with FlowJo software.

### Statistical analysis

Statistical analysis was performed with GraphPad Prism 7 software. Data were considered statistically significant if the *p*-value obtained was lower than 0.05. Data were compared with the paired or unpaired Student’s *t*-tests for values following a Gaussian distribution with similar variances. For multigroup comparisons, we applied one-way or two-way ANOVA.

### Reporting summary

Further information on research design is available in the Nature Research Reporting Summary linked to this article.

## Supplementary information

Supplementary Information

Peer Review File

Reporting Summary

Description of Additional Supplementary Files

Supplementary Movie 1

Source Data

## Data Availability

The source data underlying Figs. 1a–c, 2a, c, d, e, g, 3b, d, f, 4a–f, 5a, 5b, 6c and 7b and Supplementary Figs. 2a, b, 3a, c, 4b, c, 5a, b, 6b, 7a, d, are provided as a Source Data file. All other data are included in the supplemental information or available from the authors upon reasonable requests.
