# Supplementary material for: Tethering of vesicles to the Golgi by GMAP210 controls LAT delivery to the immune synapse
Source: Nat Commun. 2019 Jun 28;10:2864. doi: 10.1038/s41467-019-10891-w (PMC6599081; doi:10.1038/s41467-019-10891-w)
Supplement: Supplementary file 1 — Supplementary Information [file 41467_2019_10891_MOESM1_ESM.pdf]

Tethering of vesicles to the Golgi by GMAP210 controls LAT delivery to the immune synapse

Zucchetti AE et al.

SUPPLEMENTARY INFORMATION

SUPPLEMENTARY FIGURES

Supplementary figure 1

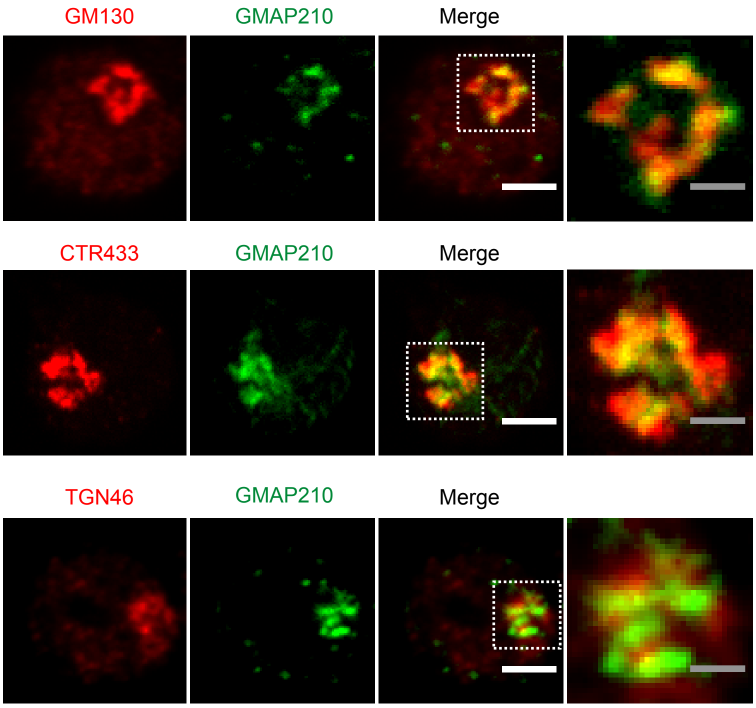

Supplementary figure 1: **GMAP-210 is present in the cis-Golgi in T lymphocytes.** Confocal images showing the relative localization of GMAP210 in red together with GM130, CTR433 and TGN46 in green. Right panel magnification of the image in the white squares. White scale bar 5µm, grey scale bar: 500nm. Images representative of two independent preparations.

Supplemantary figure 2

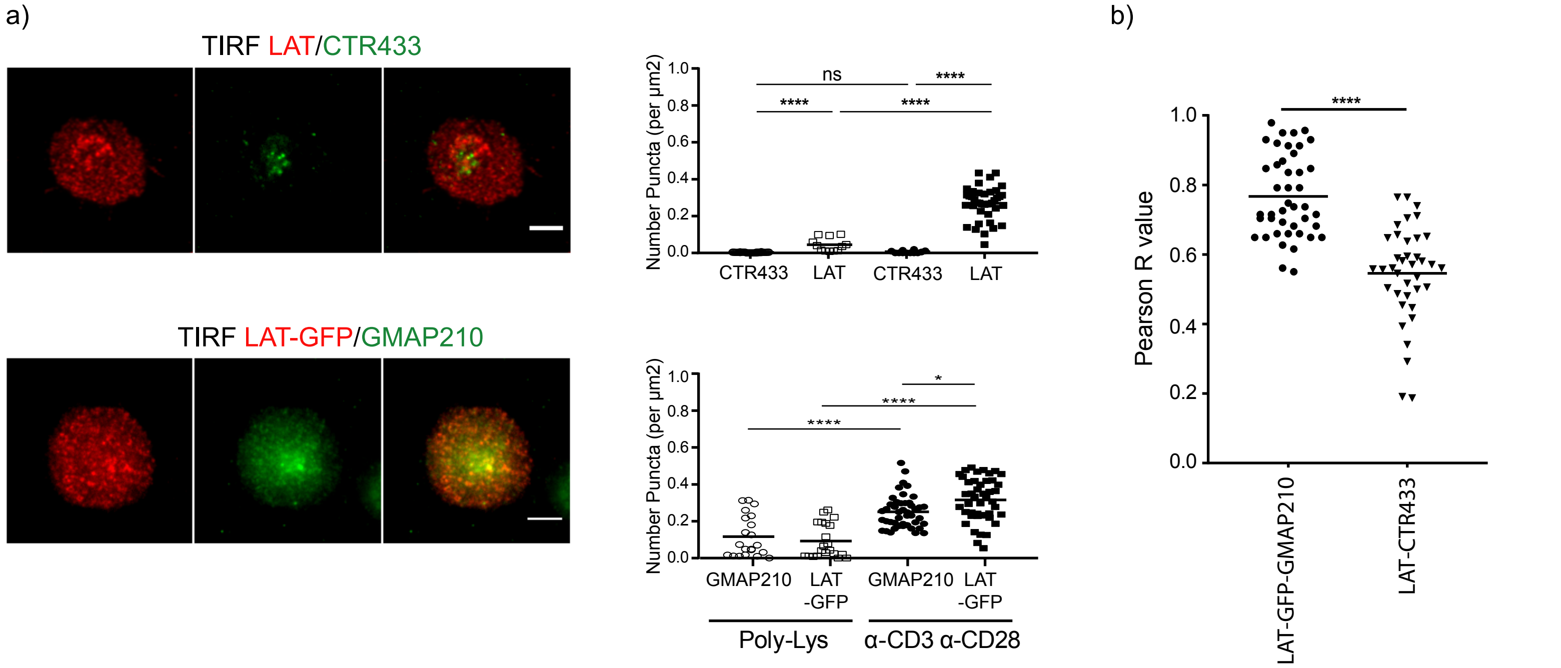

Supplementary figure 2: **GMAP210 is recruited together with LAT at the immune synapse.** a) Representative TIRF images of Jurkat cells incubated for 10 minutes on coverslips coated with anti-CD3ε+antiCD28 Abs, mimicking immune synapse formation, before fixation and staining for LAT-GFP, GMAP210, or CTR433, scale bars: 5µm. Dot plots on the right side of the panel show the quantification of the microclusters formed by the different molecules in the evanescent field expressed in microclusters/µm2 (right). Poly-L-Lysine (Poly-Lys) alone (resting conditions) or anti-CD3/CD28 (α-CD3ε α-CD28) immune synapse formation. **b**, Quantification of the colocalization of LAT-GFP with GMAP210, CTR433. Each dot = one cell; horizontal lines = median. *<P0.05, **** P<0.0001, ns: non- significant (one-way ANOVA in a t-test in b). Data represent one experiment in a and b.

Supplementary figure 3

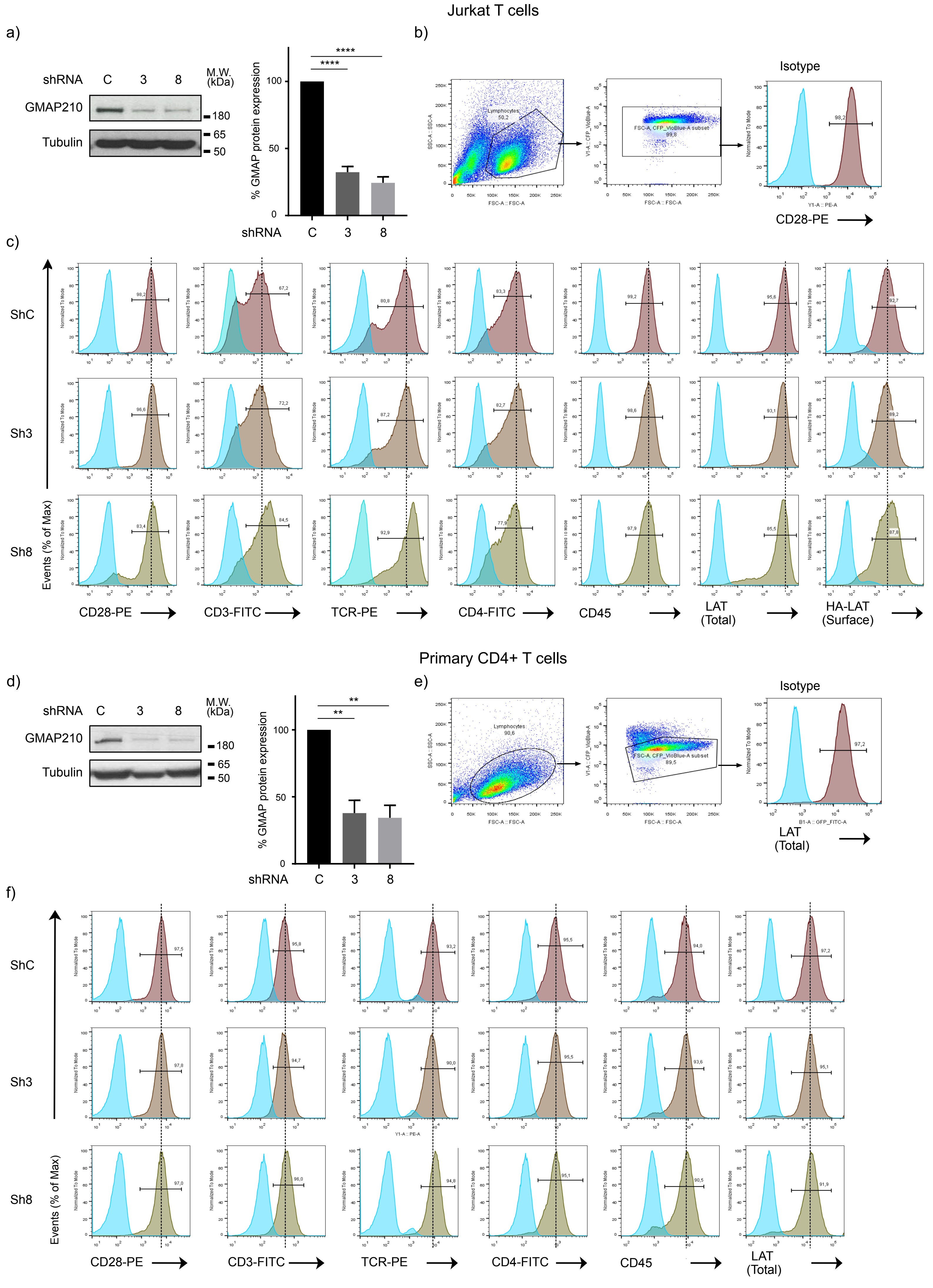

Supplementary figure 3: **GMAP-210 silencing in Jurkat and human primary T cells does not affect the expression of classical markers of T cells. a** and **d,** Immunoblot showing GMAP-210 and α-tubulin (loading control) in Jurkat (**a**) and primary T cells (**d**) expressing control (shC) or GMAP-210-specific (sh3 and sh8) shRNA and quantification of GMAP-210 expression normalized to α -tubulin expression and control shRNA. n=3 experiments. **b** and **e**,Gating strategy to determine the percentage of Jurkat (**b**) or primary CD4+ (**e**) cells expresing different classical markers. **c** and **f** Flow cytometry analysis of different markers in Jurkat (**c**) and primary T cells (**f**) expressing control and GMAP-210-specific shRNA. In blue: isotype controls. Experiment representative of three independent preparations.

Supplementary figure 4

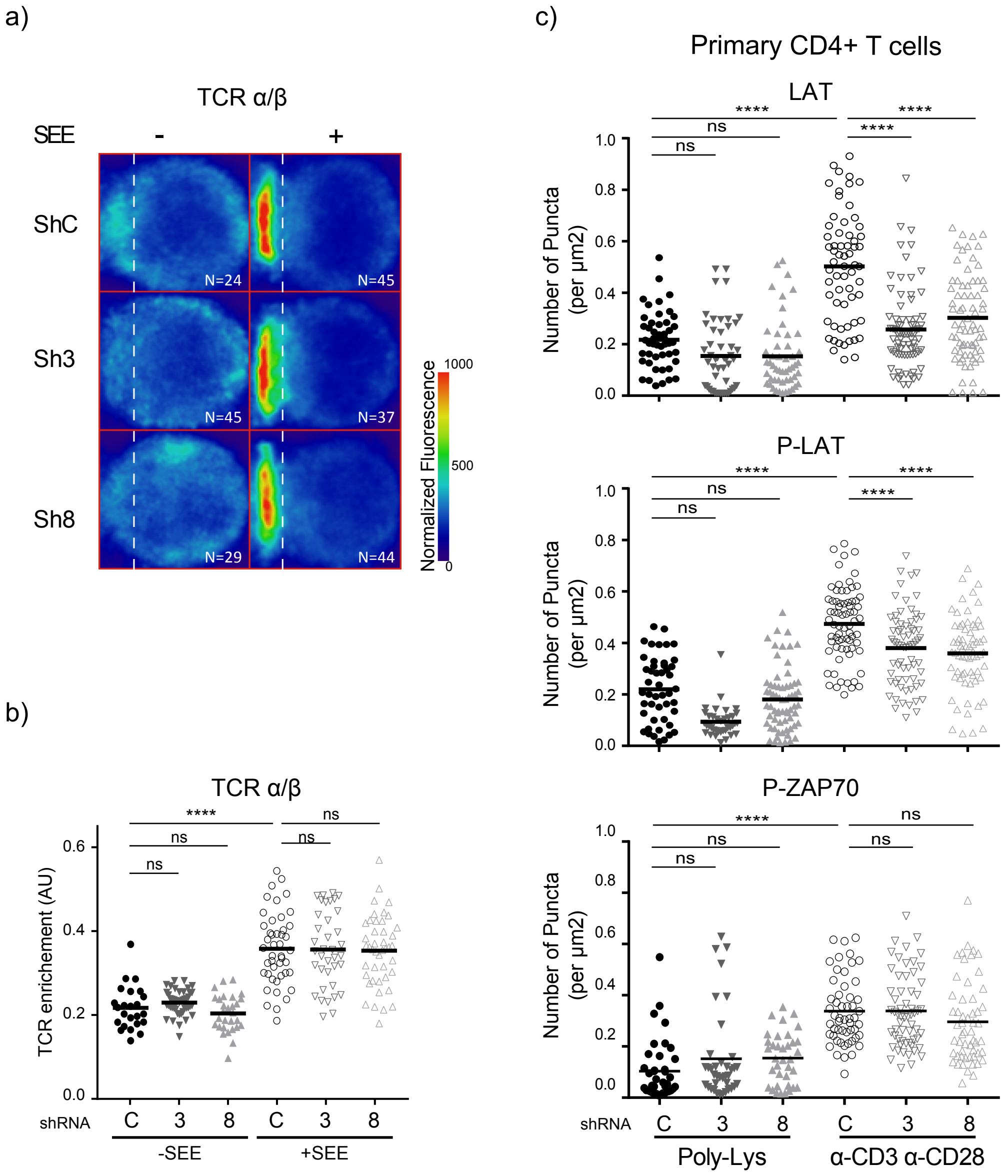

Supplementary figure 4: **GMAP-210 expression controls formation of the immune synapse. a-b**, Confocal images (**a**) and quantification (**b**) of the enrichment of the TCR at the immune synapse (depicted by the dotted white line) in Jurkat "mean cells" expressing non-targeting control shRNA (C) or GMAP-210-targeting shRNA (3 and 8) and interacting for 30min with Raji cells left unpulsed (-, unactivated state) or pulsed with SEE (+, immune synapse formation). N= number of cells constituting the mean image. (**b**) Horizontal lines represent median. **c**, Quantification of TIRF images showing the density of puncta of endogenous LAT, phosphorylated (P-) LAT, and phosphorylated (P-) ZAP70 in primary T cells expressing non-targeting control shRNA (C) or GMAP-210-specific shRNA (3, 8). Cells were incubated for 10 min on coverslips coated with poly-L-Lysine alone (resting conditions) or anti-CD3ε+antiCD28 Abs (α-CD3 α-CD28, immune synapse formation) before fixation and staining. Each dot = one cell; horizontal lines = median. **** P<0.0001, ns: non-significant (one-way ANOVA). Data represent one experiment (a and b) and three experiments (c).

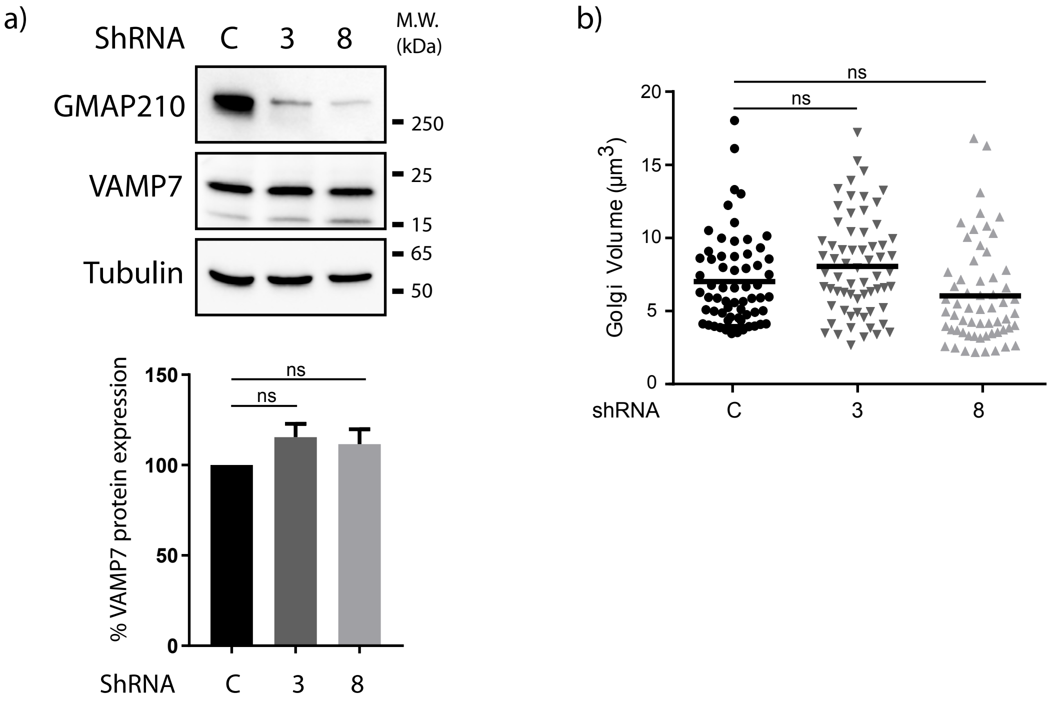

Supplementary figure 5: **VAMP7 expression and Golgi volume in Jurkat cells silenced for GMAP210. a**, Immunoblot showing GMAP210, VAMP7 and α-tubulin (loading control) in Jurkat expressing control (ShC) or GMAP210-specific (Sh3 and Sh8) ShRNA and quantification of VAMP7 expression normalized to α-tubulin expression and control shRNA. **b**, Quantification of the Golgi volume in whole cells expressing shRNAs. Each dot = one cell; horizontal lines = median. ns: non-significant (one-way ANOVA). Data represent more than three experiments in a and two independent experiments in b.

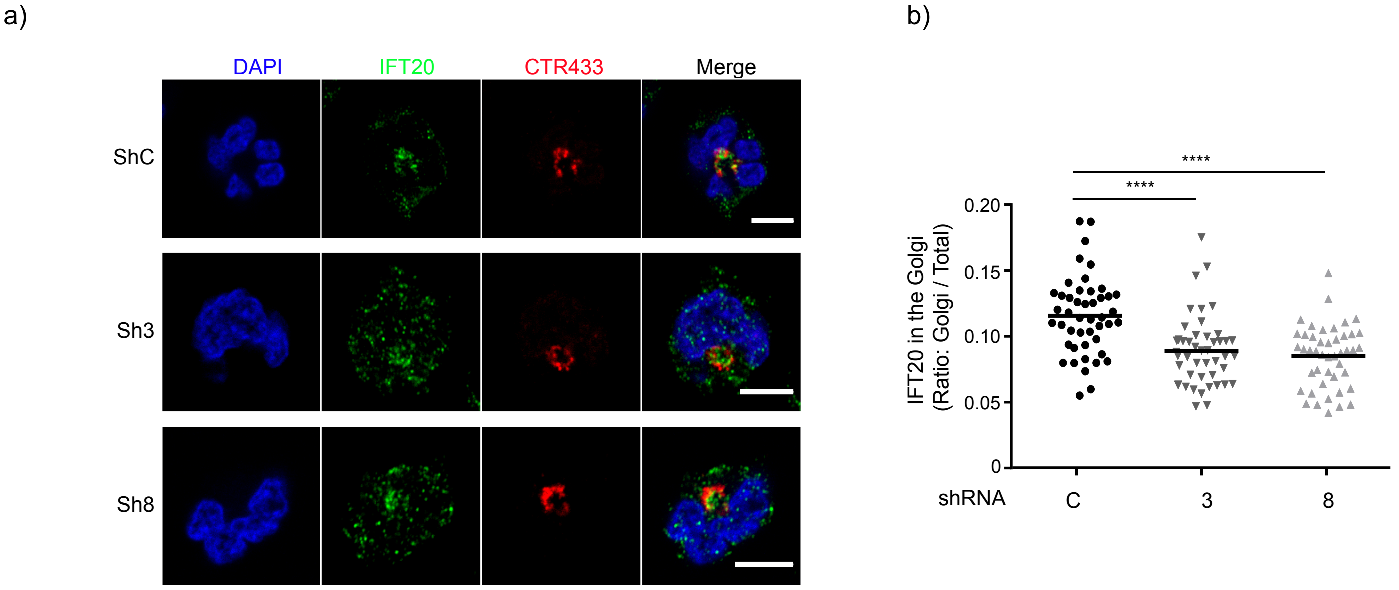

Supplementary figure 6: **IFT20 localization in Golgi in Jurkat cells silenced for GMAP210. a, b**, Confocal images showing the relative localization of IFT20 (green) and CTR433 (red) in Jurkat cells expressing control (ShC) or GMAP-210 specific shRNA (Sh3, Sh8) (nucleus in blue). **b** Quantification of the ratio of IFT20 fluorescence present in the Golgi versus total fluorescence in the different conditions. Scale bar 5μm. Each dot = one cell; horizontal lines = median. **** P<0.0001, (one-way ANOVA). Data and Images represent two independent experiments.

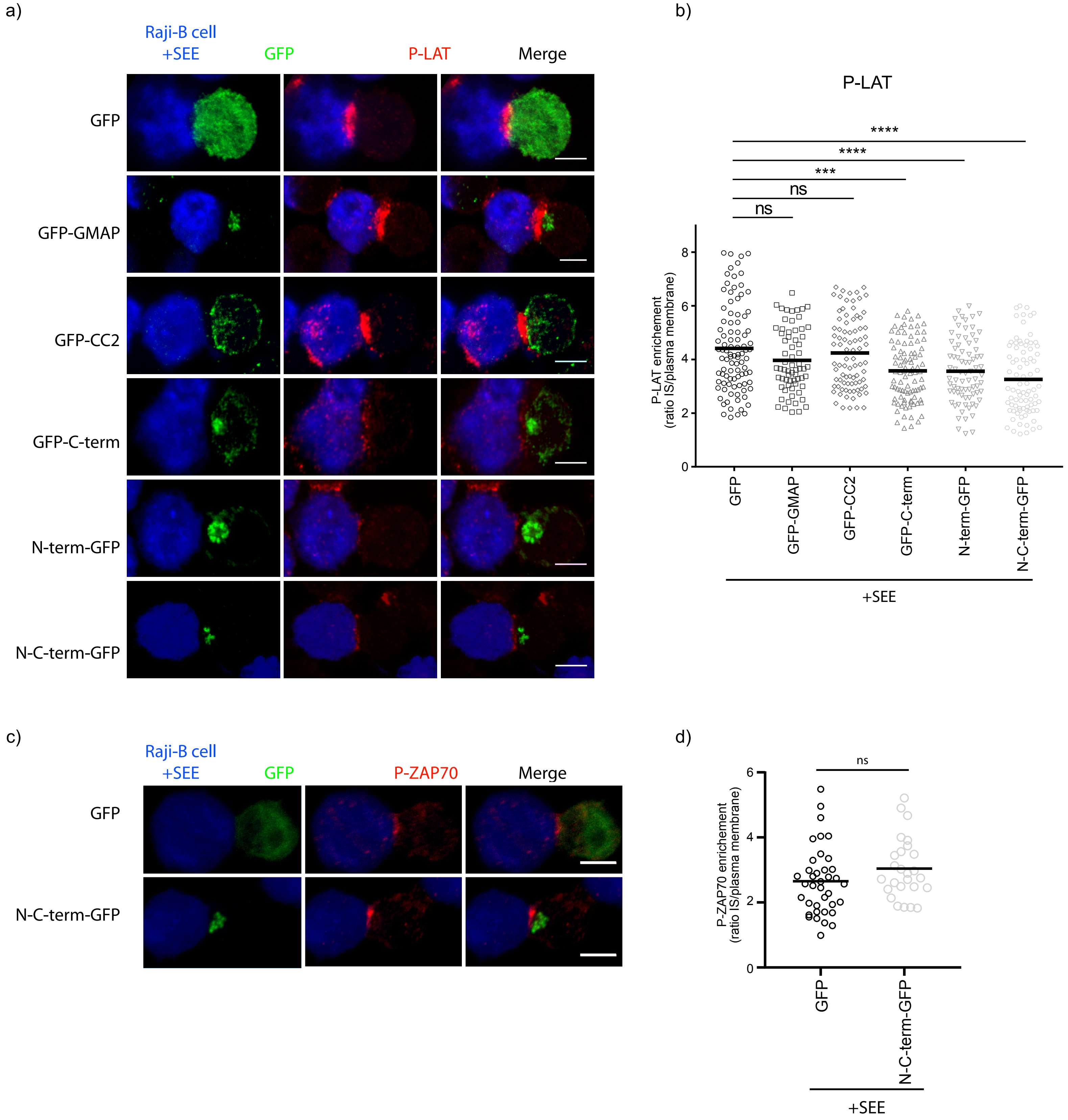

Supplementary Figure 7: **GMAP210 tethering activity controls LAT phosphorylation at the immune synapse. a-d** Confocal images of conjugates of Jurkat cells expressing GFP alone, GMAP210-GFP or different GMAP210 domains coupled to GFP and SEE-pulsed Raji B cells (in blue) labeled with anti-P-LAT (a, showed in red) or anti-P-ZAP (c, showed in red) and anti-GFP (in green) antibodies, assessed at 30 min, and quantification (b and d) of the mean fluorescence intensity of P-Lat, assessed in a fixed region of the immunological synapse and divided by the average of the mean intensities measured in three regions of the same size at the plasma membrane outside of the IS. Horizontal lines represent median. Each dot = one cell; horizontal lines = median. Scale bars= 5 µm. *** P<0.001, **** P<0.0001 ns: non-significant (ANOVA one way and parametric t-test). Data represents three experiments in b and one experiment in d.

SUPPLEMENTARY TABLES

Supplementary Table 1: List of antibodies used in this study

Primary Antibodies	Dilution IF	Dilution WB	Dilution FC	Company	Cat. N°	Assays
Rabbit anti-GMAP210	1:100	1:1000	n.a.	Gift from Michel Bornens(*)		IB: Fig 1c, 4a, 2a.<br>IF: Fig s1 (middle), 2b, 2c, 5a<br>EM: Fig 1b, 1d.
Mouse anti-GMAP210	1:25	1:250	n.a.	BD Transduction Lab	611712	IB: Fig 1a, 2f, S2a, S2c.<br>IF: 3c, Fig s1 (uper and lower).
Rabbit anti-GM130	1:100	1:1000	n.a.	Sigma-Aldrich	G7295	IB: Fig 1a, 1c, 2f. IF: FigS1
Human anti-GM130	1:100			RPAPIC (**)		IF: Fig 2d
Rabbit anti-LAT	1:100	1:1000	n.a.	Millipore	06-807	IB: Fig 1a, 1c, 2f, 4a, 2a.<br>IF: Fig 2c, 3a, 3e, 7a, 8b, S3c.<br>EM: Fig 1b 1d
Mouse anti-LAT (stock at 0,5mg/mL)	1:100	n.a.	1:100	R&D Systems	MAB 63341	IF: Fig 2b, 8a.<br>FC: Fig S2b, S2d,
Rabbit anti-Vamp7	n.a.	1:250	n.a.	Gift from Thierry Galli (***)		IB: Fig 1a, 1c, 2f, 4a, S4a.
Mouse anti-VAMP7	1:400	n.a.	n.a.	Novus Biologicals	NBP1-07118	IF: Fig 5a, 5b, S4b
Rabbit anti VAMP3	n.a.	1:100	n.a.	Gift from Thierry Galli (***)		IF: Fig 5a, 5b
Human anti-CTR433	1:50	n.a.	n.a.	RPAPIC (*)		IF: Fig 5a, 6b, S1, S2a S4a, S4d, S5a
Human anti-GFP	1:200	n.a.	n.a.	RPAPIC (*)		IF: Fig 2c, 3a, 3c, 5a, 5b, 6b, 7a, 8a, 8b, S2a, S7a, S7b.
Rabbit anti-SLP76	n.a.	1/1000	n.a.	cell signaling	4958S	IB: Fig 2f, 4a
Rabbit anti-PLC-ɔ	n.a.	1/1000	n.a.	cell signaling	2822S	IB: Fig 2f, 4a
Rabbit anti-LCK	n.a.	1/1000	n.a.	cell signaling	2787S	IB: Fig 2f, 4a
Mouse anti-CD3ζ	n.a.	1/1000	n.a.	Santa Cruz	SC-1239	IB: Fig 2f, 4a
Rabbit anti-P-LAT	1:50	n.a.	n.a.	cell signaling	3584S	IF: Fig 3c, 3e, S3c, S4c, S7a
Rabbit anti-IFT20	1:50	n.a.	n.a.	Provided by Greg Pazour		IF: Fig 6b, S4a, S4d, S5a
Rabbit anti-P-ZAP70	1:50	n.a.	n.a.	cell signaling	2701L	IF: Fig 3c, 3e, S7b
Rabbit anti-ARLB13b	1:400	n.a.	n.a.	Proteintech	17711-1-AP	IF: Fig 8a
Rabbit anti-TGN46	1:200	n.a.	n.a.	abcam	16052	IF: Fig S1
Mouse anti-Acet tubulin	1:100	n.a.	n.a.	Sigma-Aldrich	T6793	IF: Fig 8b
Mouse anti-TCRα/β PE	1:20	n.a.	1:10	Beckman Coulter	PNA39499	IF: Fig S3. FC: Fig S2b, S2d
Mouse anti-CD4 FITC	n.a.	n.a.	1:100	BD Pharmingen	555346	FC: Fig S2b, S2d
Mouse anti-CD3 FITC	n.a.	n.a.	1:100	BD Pharmingen	555332	FC: Fig S2b, S2d
Mouse anti-CD28 PE	n.a.	n.a.	1:100	BD Pharmingen	555729	FC: Fig S2b, S2d
Mouse anti CD45	n.a.	n.a.	1:100	TONBO BIOSCIENCES	70-0459-U10(	FC: Fig S2b, S2d
Rabbit anti-HA	n.a.	n.a.	1:100	cell signaling	3724S	FC: Fig S2b

Secondary Antibodies	Dilution IF	Dilution WB	Dilution FC	Company	
Directed against the species of primary antibodies and conjugated with Alexa 488, Cy3, Cy5, AMCA and HRP, raised in Donkey	1:400	1:10000		Jackson ImmunoResearch	(*)Michel Bornens Inst. Curie, Paris, France)<br>(**) RPAPIC = Recombinant Protein and Antibody Platform of the Inst. Curie<br>(***) Thierry Galli (Center of Psychiatry and Neurosciences, Paris, France)
Directed against the species of primary antibodies and conjugated with Alexa 488, Cy3, mcherry, Cy5, AMCA and HRP, raised in Goat	1:200			Invitrogen	
