# Supplementary material for: Tethering of vesicles to the Golgi by GMAP210 controls LAT delivery to the immune synapse
Source: Nat Commun. 2019 Jun 28;10:2864. doi: 10.1038/s41467-019-10891-w (PMC6599081; doi:10.1038/s41467-019-10891-w)
Supplement: Supplementary file 3 — Reporting Summary [file 41467_2019_10891_MOESM3_ESM.pdf]

nature research

Corresponding author(s): *Double-blind peer review submissions: write DBPR and your manuscript number here instead of author names.*

Last updated by author(s): *YYYY-MM-DD*

Reporting Summary

Nature Research wishes to improve the reproducibility of the work that we publish. This form provides structure for consistency and transparency in reporting. For further information on Nature Research policies, see Authors & Referees and the Editorial Policy Checklist.

Statistics

For all statistical analyses, confirm that the following items are present in the figure legend, table legend, main text, or Methods section.

n/a	Confirmed	
☐	☒	The exact sample size (*n*) for each experimental group/condition, given as a discrete number and unit of measurement
☐	☒	A statement on whether measurements were taken from distinct samples or whether the same sample was measured repeatedly
☐	☒	The statistical test(s) used AND whether they are one- or two-sided<br>*Only common tests should be described solely by name; describe more complex techniques in the Methods section.*
☐	☒	A description of all covariates tested
☐	☒	A description of any assumptions or corrections, such as tests of normality and adjustment for multiple comparisons
☐	☒	A full description of the statistical parameters including central tendency (e.g. means) or other basic estimates (e.g. regression coefficient) AND variation (e.g. standard deviation) or associated estimates of uncertainty (e.g. confidence intervals)
☐	☒	For null hypothesis testing, the test statistic (e.g. *F*, *t*, *r*) with confidence intervals, effect sizes, degrees of freedom and *P* value noted<br>*Give P values as exact values whenever suitable.*
☒	☐	For Bayesian analysis, information on the choice of priors and Markov chain Monte Carlo settings
☒	☐	For hierarchical and complex designs, identification of the appropriate level for tests and full reporting of outcomes
☒	☐	Estimates of effect sizes (e.g. Cohen's *d*, Pearson's *r*), indicating how they were calculated

Our web collection on statistics for biologists contains articles on many of the points above.

Software and code

Policy information about availability of computer code

Data collection	See material and methods. Microscopy data were collected through Metamorph (TIRF microscpy), LAS X (Leica SP8) and ZEN (LSM780, Zeiss) software. Raw data are available on request.
Data analysis	Confocal and TIRF Images were registered and cropped with Fiji (ImageJ). Fluorescence data were analyzed through Fiji and the quantification elaborated with Fiji macro language. The codes are not provided with the manuscript because not (yet) easily readable and commented but are available "as they are" on request.

For manuscripts utilizing custom algorithms or software that are central to the research but not yet described in published literature, software must be made available to editors/reviewers. We strongly encourage code deposition in a community repository (e.g. GitHub). See the Nature Research guidelines for submitting code & software for further information.

Data

Policy information about availability of data

All manuscripts must include a data availability statement. This statement should provide the following information, where applicable:

- Accession codes, unique identifiers, or web links for publicly available datasets
- A list of figures that have associated raw data
- A description of any restrictions on data availability

Some of the data generated and analyzed during this study are avalaible in the "Data source file" and the other are available under reasonable request to the corresponding author

Field-specific reporting

Please select the one below that is the best fit for your research. If you are not sure, read the appropriate sections before making your selection.

☒ Life sciences ☐ Behavioural & social sciences ☐ Ecological, evolutionary & environmental sciences

For a reference copy of the document with all sections, see nature.com/documents/nr-reporting-summary-flat.pdf

Life sciences study design

All studies must disclose on these points even when the disclosure is negative.

Sample size	No particular method has been used for the choice of the sample size. For most of experiments we chose to repeat experiments at least 3 times per condition and the number of cells available in the experiment has been maximized compatibly with the experimental setup (microscopy time, camera capabilities and cell survival).
Data exclusions	For confocal analysis data were excluded when slide preparation did not show a specific staining. We excluded experiments where no silencing was obtained.
Replication	Number of replication is indicated in the figure legends
Randomization	NA
Blinding	NA

Reporting for specific materials, systems and methods

We require information from authors about some types of materials, experimental systems and methods used in many studies. Here, indicate whether each material, system or method listed is relevant to your study. If you are not sure if a list item applies to your research, read the appropriate section before selecting a response.

Materials & experimental systems

n/a	Involved in the study	
☐	☒	Antibodies
☐	☒	Eukaryotic cell lines
☒	☐	Palaeontology
☒	☐	Animals and other organisms
☐	☒	Human research participants
☒	☐	Clinical data

Methods

n/a	Involved in the study	
☒	☐	ChIP-seq
☐	☒	Flow cytometry
☒	☐	MRI-based neuroimaging

Antibodies

Antibodies used	Antibodies used, concentration, company and catalog number are provided in supplementary table1.
Validation	See supplementary Table number 1.

Eukaryotic cell lines

Policy information about cell lines

Cell line source(s)	The source of the cell lines is indicated in Methods section
Authentication	The authentification of the cell lines is indicated in Methods section
Mycoplasma contamination	All cell lines were tested for mycoplasma (PCR test by Eurofins GATC Biotech GmbH) and were negative
Commonly misidentified lines (See ICLAC register)	NA

Human research participants

Policy information about studies involving human research participants

Population characteristics	Buffy coats from healthy donors (both male and female donors) were obtained from Etablissement Français du Sang (Paris, France) in accordance with INSERM ethical guidelines
Recruitment	Volunteer human donors
Ethics oversight	Etablissement Français du Sang and INSERM

Note that full information on the approval of the study protocol must also be provided in the manuscript.

Flow Cytometry

Plots

Confirm that:

☒ The axis labels state the marker and fluorochrome used (e.g. CD4-FITC).

☒ The axis scales are clearly visible. Include numbers along axes only for bottom left plot of group (a 'group' is an analysis of identical markers).

☐ All plots are contour plots with outliers or pseudocolor plots.

☒ A numerical value for number of cells or percentage (with statistics) is provided.

Methodology

Sample preparation	Described in materials and methods
Instrument	MACS Quant (Miltenyi)
Software	FlowJO
Cell population abundance	Cell lines and primary T-cells were used and their purity are shown in the relevant figure
Gating strategy	Gated on the lived cells (negative staining for Fixable Violet Dead Cell)

☒ Tick this box to confirm that a figure exemplifying the gating strategy is provided in the Supplementary Information.
