# Supplementary material for: Tethering of vesicles to the Golgi by GMAP210 controls LAT delivery to the immune synapse
Source: Nat Commun. 2019 Jun 28;10:2864. doi: 10.1038/s41467-019-10891-w (PMC6599081; doi:10.1038/s41467-019-10891-w)
Supplement: Supplementary file 4 — Description of Additional Supplementary Files [file 41467_2019_10891_MOESM4_ESM.pdf]

Description of Additional Supplementary Files

Supplementary Movie 1:
GMAP210 is recruited together with LAT at the immune synapse. Time-lapse TIRF microscopy of Jurkat cells co-transfected with Lat-mCherry (red) and GMAP210-GFP (green) seeded on coverslips coated with anti-CD3ε +antiCD28 Abs. Left: Lat-mCherry, middle: GMAP210-GFP, right: merge. Scale bar 5µm."
