# Supplementary material for: Tethering of vesicles to the Golgi by GMAP210 controls LAT delivery to the immune synapse
Source: Nat Commun. 2019 Jun 28;10:2864. doi: 10.1038/s41467-019-10891-w (PMC6599081; doi:10.1038/s41467-019-10891-w)
Supplement: Supplementary file 2 — Peer Review File [file 41467_2019_10891_MOESM2_ESM.pdf]

Reviewers' comments:

Reviewer #1 (Remarks to the Author):

The authors have investigated the role of the Golgi tethering protein GMAP210 on LAT localization and function in the immunological synapse. They use biochemical, subcellular fractionation and microscopy (EM and light) to investigate this in Jurkat T cells subjected to shRNA and plasmid based protein expression to manipulate the system. Fragments of GMAP210 that act as dominant negatives reduce the efficiency of LAT recruitment and amplitude of T cell activation. Experiments are done in both Jurkats and primary T cells. GMAP210 and LAT localize to primary Cilium in epithelial cells. The study establishes an analogy between the TGN and the primary cilium which is leverage for T cell signling.

Specific concerns-
The data are generally convincing, but the enrichment ratio is not the best parameter to quantify pLAT. First, based on images in Figure 7 its unclear to me how total LAT and pLAT can have similar enrichment ratios as the apparent ratio of signals is very different if I understand the measurement. The enrichment ratio make sense for total LAT, but for pLAT, which should only exist in the synapse if the signal is specific it seems like a different ratio should be compared on a single cell level- perhaps a ratio between pLAT and pZAP-70 or something of this nature that would relate to efficiency of LAT activation in relation to early TCR signaling.

Reviewer #2 (Remarks to the Author):

Tethering of vesicles to the Golgi by GMAP210 controls LAT delivery to the immune synapse

Zucchetti et al.
Manuscript ID: NCOMMS-18-34369

In this manuscript, the authors describe the colocalization of the Golgin GMAP210 with intracellular LAT vesicles and recruitment of those vesicles to the immune synapse upon stimulation. Furthermore, using siRNA they demonstrate a role for GMAP210 in LAT recruitment and phosphorylation and T cell activation. They also identify the domains in GMAP210 involved in LAT recruitment. From these experiments, they propose that GMAP210 captures LAT vesicles that have been retrotransported to the golgi and directs them to the immune synapse.

Overall, the experiments in this study are well-performed, well-controlled and the insights about LAT trafficking and the role for GMAP210 are novel. However, there are some discrepancies in the observations and the proposed model that should be clarified.

1. The authors show co-fractionation (Fig. 1a) and colocalization by EM (Fig. 2a) of LAT and GMAP210 in unstimulated cells. But according to their model (and based on confocal imaging), GMAP210 interacts with LAT vesicles internalized upon stimulation. The authors should clarify this discrepancy. They could look at whether colocalization by fractionation and EM increases in stimulated cells.

2. In the same vein, authors should show the Pearson r value in 2d between GMAP and LAT in unstimulated cells to see whether an increase in colocalization between the two molecules is evident in this assay.
Minor points: They should also include Pearson r values for confocal images in 2b. Additionally, the difference between the colocalization of GMAP210 and LAT in a Raji+SEE induced synapse (2b – very

little coloc) and a cell stimulated on a coverslip (2c – very high coloc) is striking (2c). Could this be the difference between antibody staining of GMAP210 (2b) and GMAP210-GFP overexpression? Authors should clarify.

3. Similarly, in Figure 5, the authors capture VAMP7 expressing vesicles with GMAP210 tethered to mitochondria in unstimulated cells. The authors should clarify their model based on the observation of GMAP210 interaction with LAT and VAMP7 in unstimulated cells. Perhaps what they are seeing is a low level of steady state interactions between these molecules that does not have a quantifiable impact upon GMAP210 silencing on steady state LAT trafficking. But then authors should demonstrate increased coloc upon stim (see comments 1 and 2 above).

4. The authors should look at a microcluster marker such as phosphotyrosine or pLAT or ZAP70 in the fixed cells in Figure 2c to ensure that what they are quantifying as microclusters are indeed microclusters. This is especially important as they are looking at LAT at 10min after stimulation and a recent paper (Balagopalan et al., Nat Comm 2018) showed that the major wave of LAT microclusters are gone by this time. Adding a microcluster marker to their analysis would clarify this.

5. Strikingly, the colocalization of GMAP210-GFP and LAT-mcherry in the TIRF video movie appears to be completely on vesicles moving on microtubules, not microclusters. It appears that the TIRF angle is not stringent enough, as we can see the outline of the entire cell as it retracts and moves on the coverslip. If the authors want to demonstrate specific delivery of LAT and GMAP vesicles to microclusters, they should add a microcluster marker such as ZAP70 to their live cell imaging studies and do three color imaging or at least pair-wise imaging. It would also be useful to see live cell imaging of LAT and GMAP210 in unstimulated conditions.

6. Minor point: In Figure 4a the authors should comment on the differences between siRNA 3 and 8 in LAT recruitment to the beads. Moreover, quantification of the blots should be provided.

Reviewer #3 (Remarks to the Author):

In this interesting study, the authors extend their previous, original analysis of the mechanisms that regulate LAT transport to the IS. Using imaging and biochemical analyses, they demonstrate that GMAP210 is present in LAT-containing vesicles, and colocalizes with it to the IS. They go on to demonstrate that siRNA-mediated silencing go GMAP210 specifically blocks LAT recruitment and phosphorylation at the IS,the formation of a LAT-dependent signaling platform, and T cell activation measured by cytokine expression. In addition, GMAP210 captures vesicles carrying the VAMP7 vSNARE
known to control LAT trafficking, and it is able to localize LAT to the cilium (which displays some similarity to the IS) in ciliated cells.

The technical quality of this study is high, and the results generally support the conclusion, and improve our understanding of the processes controlling LAT IS translocation and T cell activation. There are, however, several mild concerns that need to be addressed by the authors:

1) Fig. 4a: While both shRNAs are equally effective in knocking down GMAP210 expression and reducing th membrane expression of PLCg and SLP-76, sh8 is not very effective in reducing LAT expression. Some explanation is needed.

2) Fig. 4a: The authors' statement that GMAP210 silencing resulted in less VAMP7 in the "signaling

complexes" is not convincing since the reduction in the VAMP7 signal is minimal. This is particularly worrisome since the legend states that this result is representative of >3 experiments. Certainly, the later statement (p. 11) that "VAMP7 was not recruited to the LAT signalosome when GMAP210 was silenced (figure 4a)" is a stretch of the imagination. Quantitation of the VAMP7 signal (normalized to CD3-zeta level should be performed.

3) The authors should explain why they observe 2 VAMP7 bands in the "signaling complexes", only one band is present in the input material (Fig. 4a).

4) The specificity of GMAP210 for vesicles delivered to the IS (containing GMAP210 but not TCR-zetamentioned in the Discussion (p. 17) could potentially be explained by TCR-zeta being present in larger vesicles with a lower curvature. Since the authors can distinguish between "small" and "large" vesicles (Fig. 2a) it would be interesting to determine if, indeed, TCR-zeta is found in the "large" vesicles seen in Fig. 2a.

Manuscript NCOMMS-18-34369, Point by point answer to reviewers.

We would first like to thank the editor and reviewers for the time they spent to critically analyze our work and their constructive comments. Their comments were indeed very precious to improve the quality of our work.

Reviewer #1 (Remarks to the Author):

Specific concerns:

"The data are generally convincing, but the enrichment ratio is not the best parameter to quantify pLAT. First, based on images in Figure 7 its unclear to me how total LAT and pLAT can have similar enrichment ratios as the apparent ratio of signals is very different if I understand the measurement. The enrichment ratio make sense for total LAT, but for pLAT, which should only exist in the synapse if the signal is specific it seems like a different ratio should be compared on a single cell level- perhaps a ratio between pLAT and pZAP-70 or something of this nature that would relate to efficiency of LAT activation in relation to early TCR signaling."

We thank the reviewer for this comment. We agree with the reviewer that this analysis is confusing for "markers", which appear only after activation and are normally only present at the immune synapse, i.e. pLAT and pZAP70 especially in "non-activating" conditions (-SEE).

We thus perform a new analysis on the RAW images using a methodology already used by others and us [1,2]. Briefly, in each cell, the mean fluorescence intensity at the IS of P-LAT or P-ZAP-70 was divided by the average of the mean intensities measured in three regions of the same size at the plasma membrane outside of the IS (IS-to-membrane ratio) on non normalized images. This IS-to-membrane ratio can go from 1, if only pLAT "background fluorescence" is present at the immune synapse, to 8 (in our experimental conditions) when the fluorescence measured in the synapse is 8 fold superior to the background fluorescence measured elsewhere at the membrane. Although, phosphoproteins are not supposed to be outside of the immune synapse (as rightly pointed by the reviewer), comparing ratios of fluorescence instead of fluorescence intensities allows to "normalize" variations due to the fact that the numerous images taken for the different experimental groups cannot be taken the same day (6 conditions for figure 3 and 14 conditions in figure 7). We now present the results only in activating conditions since as stated by the reviewer this analysis is not pertinent in non activated cells (new figure 4 and new supplemental figure 7c

and d). This issue also precludes the analysis proposed by the reviewer, i.e. to compare "*between pLAT and pZAP-70 or something of this nature*". Indeed, this would mean taking images in two different fluorescence channels with laser power and detector gain that might differ when we take images (numerous experimental conditions that precludes analysis the same day). We would thus need to do ratio of fluorescence in the two different channels and a ratio of these ratios, which we think is not correct. We have analyzed numerous images (at least 65 images per experimental condition) for LAT and pLAT, which give a robust statistical analysis. We thus think that our results are solid enough to say that the tethering activity of GMAP210 is required to induce LAT recruitment. To show that the tethering activity of GMAP210 does not affect "earlier" signals, we have now quantified phospho-ZAP70 at the immune synapse in cells overexpressing the short version of GMAP210. In this condition, LAT recruitment to the immune synapse and LAT phosphorylation are decreased, whereas phosphorylation of ZAP70 is not affected. These new results are presented in supplemental figure 7 c and d.

Reviewer #2 (Remarks to the Author):

1. The authors show co-fractionation (Fig. 1a) and colocalization by EM (Fig. 2a) of LAT and GMAP210 in unstimulated cells. But according to their model (and based on confocal imaging), GMAP210 interacts with LAT vesicles internalized upon stimulation. The authors should clarify this discrepancy. They could look at whether colocalization by fractionation and EM increases in stimulated cells.

We thank the reviewer for this comment, which led us to look more carefully at the presence of GMAP210 on LAT vesicles in activated cells.

To show if GMAP210 associates more with LAT-containing vesicles upon activation, we performed one of the experiments suggested by the reviewer: fractionation followed by pull down assay of vesicles containing-LAT in activating condition, which is easier to quantify than EM images. The results of this experiment show unequivocally that the interaction of GMAP210 with LAT-containing vesicles is increased upon TCR stimulation. This result is presented now in the new version of Figure 2 and we also clarified this point in the text.

2. In the same vein, authors should show the Pearson r value in 2d between GMAP and LAT in unstimulated cells to see whether an increase in colocalization between the two molecules is evident in this assay.

Minor points: They should also include Pearson r values for confocal images in 2b.

Additionally, the differences between the colocalization of GMAP210 and LAT in a Raji+SEE induced synapse (2b – very little coloc) and a cell stimulated on a coverslip (2c – very high coloc) is striking (2c).

Could this be the difference between antibody staining of GMAP210 (2b) and GMAP210-GFP overexpression? Authors should clarify.

As stated by the reviewer, the colocalization we observed was more striking on coverslips than in "conjugates". One of the differences between the models was the kinetic we used. On coverslips coated with activating Abs, images were taken after 10 min of contact, whereas images of conjugates (Jurkat-Raji) were taken 30 min after interaction between the two cells.

To explore whether the difference in the colocalization between GMAP210 and LAT could be related to this difference in kinetic, we followed and quantified GMAP210/LAT colocalization in conjugates at different time-points after activation (0, 5, 10, 15 and 30 min). We found that GMAP/LAT colocalization follows a kinetic with a maximum of colocalization at 10 min. At 30 min, colocalization of GMAP210 with LAT decreases. These results can explain the differences observed in our two models. These results are shown in the new version of the manuscript in Figure 2.

These results together with the data obtained on the purification of LAT vesicles strongly suggest that GMAP210 interaction with LAT vesicles increases with activation of T cells and follows a kinetic.

As requested by the reviewer, we have quantified the Pearson r value for the colocalization of GMAP210 and LAT in TIRFM images of non-stimulated T-cells seeded on poly-L-lysine coated slides. This value is significantly smaller in non-activated conditions than in activating conditions (see figure 1 below). However, in our hands cells do not spread on poly-L-lysine and thus the Pearson r value is strongly influenced by the background. We are thus not sure of its accuracy. We thus provide it to the reviewer but did not include it in the new manuscript.

Finally, as requested by the reviewer and to rule out the possibility that the colocalization of LAT with GMAP210 was an artefact of GMAP-GFP overexpression, we measured the Pearson r

values between the endogenous GMAP210 and LAT-GFP. The Pearson r values shown in figure 1 below, show that CD3+CD28 activation increases the colocalization between endogeneous GMAP210 and LAT-GFP. Our results also confirmed that the endogenous GMAP210 is recruited in the evanescence region under activation condition. This new data is presented in the new supplemental figure 2b. Altogether these results show that the recruitment of the endogeneous GMAP210 to the immune synapse can be observed by TIRFM.

1)

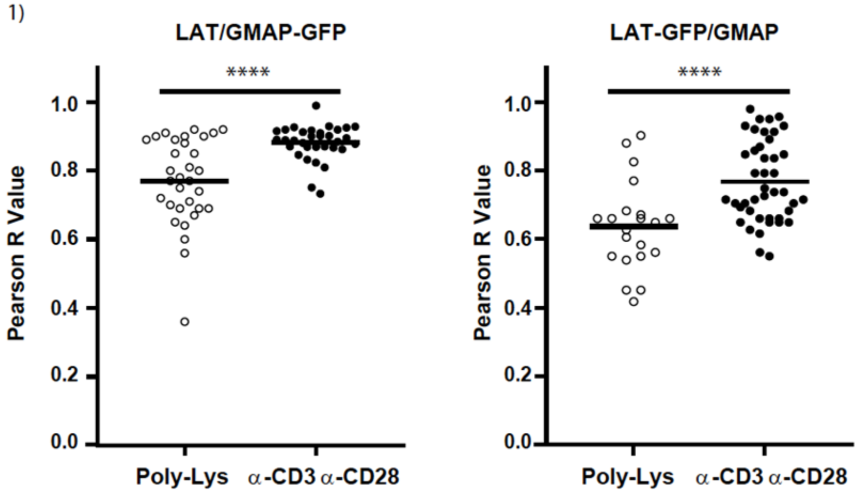

GMAP210 is recruited together with LAT at the immune synapse. Quantification of the colocalization of GMAP-GFP with LAT endogenous (left) and LAT-GFP with GMAP210 endogenous in non-stimulated (poly-Lys) or activated in coverslip coated with anti-CD3/anti-CD28. Each dot = one cell; horizontal lines = median. **** $P<0.0001$, (parametric t-test). Data represent one experiment.

3. Similarly, in Figure 5, the authors capture VAMP7 expressing vesicles with GMAP210 tethered to mitochondria in unstimulated cells. The authors should clarify their model based on the observation of GMAP210 interaction with LAT and VAMP7 in unstimulated cells. Perhaps what have a quantifiable impact upon GMAP210 silencing on steady state LAT trafficking. But then authors should demonstrate increased coloc upon stim (see comments 1 and 2 above).

The capture assay, performed in our study, requires the depolymerization of microtubules in order to scatter the Golgi into ministacks throughout the cytoplasm and thus in the proximity of many more mitochondria [3,4]. It is thus not possible to perform this assay in activating conditions since polymerization of microtubules control T lymphocyte activation [5].

Although we cannot perform the capture assay in activating conditions, we think that we now convincingly show that T lymphocyte activation is accompanied by an increase in colocalization between GMAP210 and LAT (see comments above). The capture assay reported in our study

was essentially performed to evidence the capacity of GMAP210 to bind vesicles "decorated" with the VAMP7 vesicular SNARE, which has been associated by others and us to LAT containing vesicles recruited to the immune synapse [1,6,7]. Our results show that indeed GMAP210 can bind these vesicles and redirect them to mitochondria in the capture assay.
We now clarified the goal of the assay in the new manuscript.

4. *The authors should look at a microcluster marker such as phosphotyrosine or pLAT or ZAP70 in the fixed cells in Figure 2c to ensure that what they are quantifying as microclusters are indeed microclusters. This is especially important as they are looking at LAT at 10min after stimulation and a recent paper (Balagopalan et al., Nat Comm 2018) showed that the major wave of LAT microclusters are gone by this time. Adding a microcluster marker to their analysis would clarify this.*
We realized that the term microclusters can be misleading for the readers. Indeed, some authors called "microclusters" clusters of signaling molecules present at the plasma membrane and presenting some signs of activation (recruitment of protein partners of phosphorylation). We used the micro cluster term because what we quantified on the TIRFM images was micrometric clusters of LAT, GMAP210 or other markers. Yet, in our conditions, because we are using TIRFM that cannot discriminate between the presence of fluorescent signal at the plasma membrane and in the 200 to 300nm beneath, we changed "microclusters" for puncta and clarified what we called puncta in the new version of the manuscript. The purpose was not to state that GMAP210 is present at the plasma membrane together with the microclusters (as defined above). Indeed, we rather favor the fact that GMAP210 co-localizes with vesicular pool of LAT as stated along the manuscript and proposed in the graphical model (figure 8). We now changed GMAP210 "microclusters" in the text for GMAP210 puncta.

5. Strikingly, the colocalization of GMAP210-GFP and LAT-mcherry in the TIRF video movie appears to be completely on vesicles moving on microtubules, not microclusters. It appears that the TIRF angle is not stringent enough, as we can see the outline of the entire cell as it retracts and moves on the coverslip.
We agree with the reviewer that GMAP210 and LAT might move together on vesicles rather than in microclusters situated at the plasma membrane (see answer to point 4 and below). Our model

indeed proposes that GMAP210 captures vesicles formed at the Golgi upon TCR activation and brings them close to the immune synapse. We cannot exclude that in this live imaging experiment the TIRF angle was not stringent all along the movie. Yet, we think it is reasonable to conclude that most of the events we observed represent LAT-containing vesicles bound to GMAP210 present in the 200 to 300 nm, just below the plasma membrane, in the evanescent field.

If the authors want to demonstrate specific delivery of LAT and GMAP vesicles to microclusters, they should add a microcluster marker such as ZAP70 to their live cell imaging studies and do three color imaging or at least pair-wise imaging. It would also be useful to see live cell imaging of LAT and GMAP210 in unstimulated conditions.

As stated in answer to point 4 of reviewer 2, we were probably unclear on what we called microclusters and have now changed this term throughout the manuscript (see answer to point 4). Our purpose is not to show that GMAP210 participates to the formation of microclusters at the plasma membrane (GMAP210 was indeed never observed in the plasma membrane) but rather to show that GMAP210 plays a role in the trafficking of vesicular pools of LAT recruited to the immune synapse at "later" time points as described in [7]. Our new data showing the kinetic of recruitment of GMAP210 to the immune synapse, which starts 5 minutes after activation, confirm that GMAP210 is not involved in the early formation of LAT microclusters. It rather participates to the recruitment of the second "later" wave of LAT recruitment to the synapse, which also participates in signalosome formation as shown by results presented in figure 4.

The reviewer is correct; our results as they are do not demonstrate that the delivery of vesicles by GMAP210 happens at specific places at the plasma membrane. We did not claim other wise in the previous version of our manuscript and in our graphical model. We have been especially careful not to say that we demonstrate such a thing in the new version of the manuscript. Such a demonstration would require a more resolutive microscopy with appropriate markers and would be beyond the scope of our study.

6. Minor point: In Figure 4a the authors should comment on the differences between siRNA 3 and 8 in LAT recruitment to the beads. Moreover, quantification of the blots should be provided.

As asked by the reviewer, we now show the quantifications of different experiments. These quantifications do not show any significant difference between ShRNA3 and ShRNA8 that both

significantly decreased the recruitment of signaling proteins in the signalosome. We have represented the results obtained for each experiment so that the readers can appreciate the decrease in the recruitment of LAT and other signaling molecules on the activating beads. These quantifications are presented in the new version of figure 4 (figure 4b).

Reviewer #3 (Remarks to the Author):

In this interesting study, the authors extend their previous, original analysis of the mechanisms that regulate LAT transport to the IS. Using imaging and biochemical analyses, they demonstrate that GMAP210 is present in LAT-containing vesicles, and colocalizes with it to the IS. They go on to demonstrate that siRNA-mediated silencing go GMAP210 specifically blocks LAT recruitment and phosphorylation at the IS,the formation of a LAT-dependent signaling platform, and T cell activation measured by cytokine expression. In addition, GMAP210 captures vesicles carrying the VAMP7 vSNARE known to control LAT trafficking, and it is able to localize LAT to the cilium (which displays some similarity to the IS) in ciliated cells.

The technical quality of this study is high, and the results generally support the conclusion, and improve our understanding of the processes controlling LAT IS translocation and T cell activation. There are, however, several mild concerns that need to be addressed by the authors:

1) Fig. 4a: While both shRNAs are equally effective in knocking down GMAP210 expression and reducing th membrane expression of PLCg and SLP-76, sh8 is not very effective in reducing LAT expression. Some explanation is needed.

As stated above in response to point 6 from reviewer 2, we now added the quantification of different experiments showing the decreased recruitment of LAT and other signaling molecules in the "signalosome". These quantifications do not show any significant difference between the two shRNA targeting GMAP210. Yet, they confirm that the decreased expression of GMAP210 alter the formation of the signalosome. These quantifications are presented in the new figure 4b, they show the decreased recruitment of LAT and signaling molecules for individual experiment.

2) Fig. 4a: The authors' statement that GMAP210 silencing resulted in less VAMP7 in the "signaling complexes" is not convincing since the reduction in the VAMP7 signal is minimal. This is particularly worrisome since the legend states that this result is representative of >3

experiments. Certainly, the later statement (p. 11) that "VAMP7 was not recruited to the LAT signalosome when GMAP210 was silenced (figure 4a)" is a stretch of the imagination. Quantitation of the VAMP7 signal (normalized to CD3-zeta level) should be performed.

As stated above, we have now quantified the VAMP7 signal found in the “signalosome” and normalized it to CD3-zeta signal. Results shown in the new figure 4b, confirm that VAMP7 recruitment is decreased when GMAP210 is silenced (3 experiments).

We do agree with reviewer 3 that it was not correct to state “VAMP7 was not recruited to the signalosome when GMAP210 was silenced”. We now changed the sentence to state “VAMP7 recruitment was decreased”.

3) The authors should explain why they observe 2 VAMP7 bands in the "signaling complexes", only one band is present in the input material (Fig. 4a).

As stated by the reviewer, two bands are observed in the signaling complexes. These two bands are also observed in the LAT-containing vesicles purified from the membrane floatation gradient (figure 1c). The lowest band is matching the molecular weight of VAMP7 (around 25 kDa) and corresponds to the band observed in the total cell lysate. We think that the highest band may evidence “enrichment”, in the LAT-containing vesicles, of a pool of VAMP7 presenting post-translational modifications leading to a shift of molecular weight (i.e., lipid modification or phosphorylation). In line with this hypothesis, VAMP7 has been shown to be phosphorylated [8]. Moreover, SNARE proteins containing a Longin domain, like VAMP7, are modified by lipids [9], which can also change the apparent molecular weight of proteins.

Although this interesting point should be addressed, we think that studying the posttranslational modifications of VAMP7 is beyond the scope of our study. We added a comment on the potential signification of these two bands in the new version of the manuscript.

4) The specificity of GMAP210 for vesicles delivered to the IS (containing GMAP210 but not TCR-zetamentioned in the Discussion (p. 17) could potentially be explained by TCR-zeta being present in larger vesicles with a lower curvature. Since the authors can distinguish between "small" and "large" vesicles (Fig. 2a) it would be interesting to determine if, indeed, TCR-zeta is found in the "large" vesicles seen in Fig. 2a.

We thank the reviewer for this useful suggestion. To answer the reviewer, we performed EM on Jurkat cells expressing CD3-□-GFP and labeled both GMAP210 and CD3-□. We noticed that a large proportion of CD3-□ was present in multivesicular bodies (MVB) and that little co-localization of CD3-□ with GMAP210 was observed. GMAP210, as expected and reported in the first version of our manuscript, was present on small vesicles around the Golgi (see figure 2 below). These results are in agreement with studies including ours showing the presence of the CD3 complexes on exosomes that are generated inside MVB [10]. Of note MVB are bigger than the vesicles shown to bear LAT. They should thus not be taken in charge by GMAP210 for their traffic, which fits with our results. Because we did not succeed to label LAT, CD3-□ and GMAP210 together by EM, we did not include these last results in the manuscript but comment this point in the discussion.

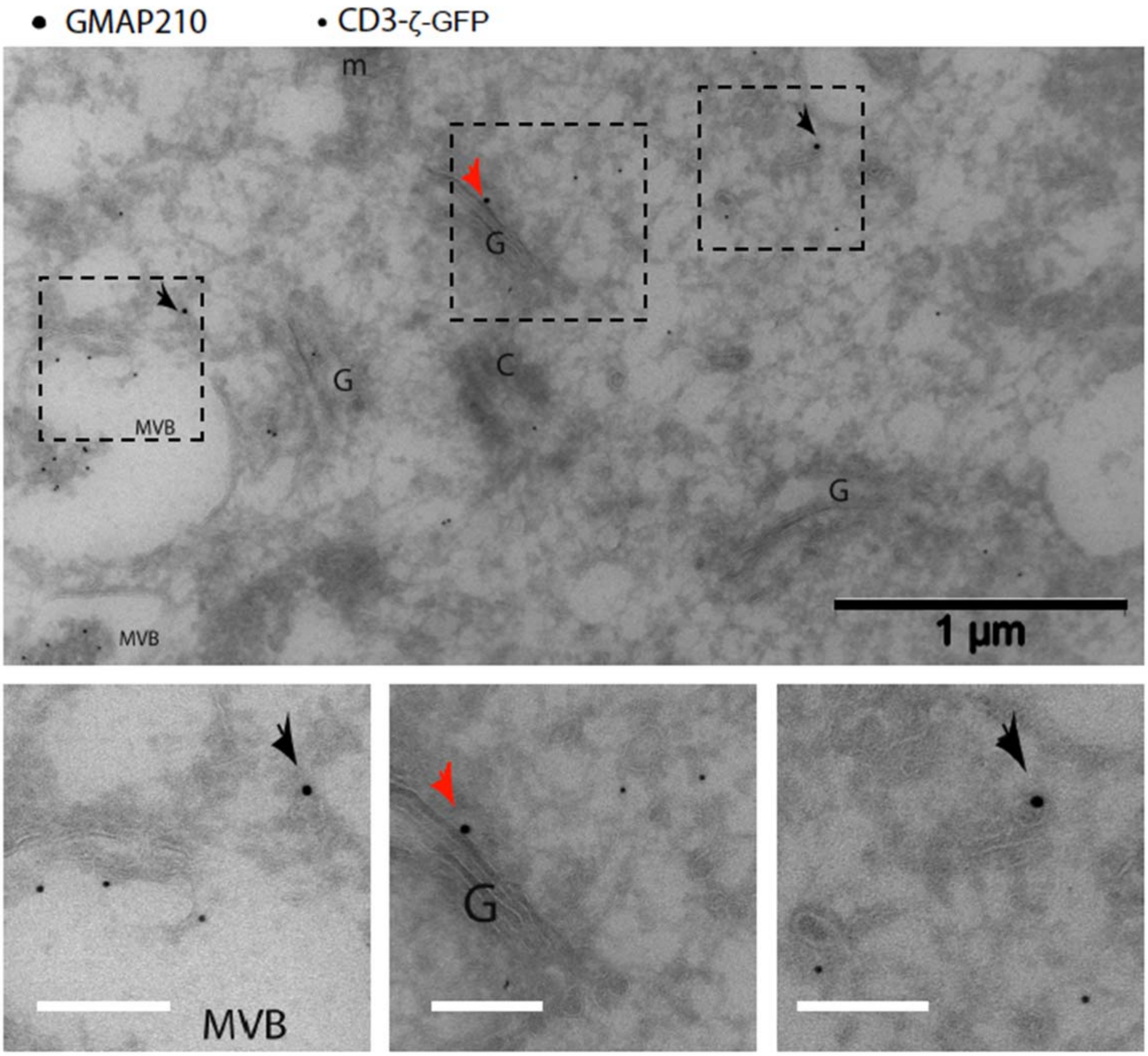

GMAP210 and CD3-ζ are present in different intracellular compartments. Transmission electron microscopy images of fixed Jurkat cells overexpressing CD3-ζ-GFP showing an immunogold staining for GFP (6nm gold particles) and GMAP210 (10nm gold particles); C, centriole; G, Golgi apparatus; MVB: Multivesicular bodies showing CD3-ζ labeling. Black arrows show small vesicles presenting GMAP210 staining, red arrow show GMAP present in the Golgi apparatus. Black scale bar: 1µm, white scale bar: 200nm.

References

1 Larghi, P. *et al.* VAMP7 controls T cell activation by regulating the recruitment and phosphorylation of vesicular Lat at TCR-activation sites. *Nat Immunol* **14**, 723-731, doi:10.1038/ni.2609 (2013).

2 Galgano, D. *et al.* The T cell IFT20 interactome reveals new players in immune synapse assembly. *Journal of cell science* **130**, 1110-1121, doi:10.1242/jcs.200006 (2017).

3 Wong, M. & Munro, S. Membrane trafficking. The specificity of vesicle traffic to the Golgi is encoded in the golgin coiled-coil proteins. *Science* **346**, 1256898, doi:10.1126/science.1256898 (2014).

4 Sato, K., Roboti, P., Mironov, A. A. & Lowe, M. Coupling of vesicle tethering and Rab binding is required for in vivo functionality of the golgin GMAP-210. *Molecular biology of the cell* **26**, 537-553, doi:10.1091/mbc.E14-10-1450 (2015).

5 Martin-Cofreces, N. B. *et al.* End-binding protein 1 controls signal propagation from the T cell receptor. *The EMBO journal*, doi:10.1038/emboj.2012.242 (2012).

6 Soares, H. *et al.* Regulated vesicle fusion generates signaling nanoterritories that control T cell activation at the immunological synapse. *The Journal of experimental medicine* **210**, 2415-2433, doi:10.1084/jem.20130150 (2013).

7 Balagopalan, L. *et al.* Plasma membrane LAT activation precedes vesicular recruitment defining two phases of early T-cell activation. *Nature communications* **9**, 2013, doi:10.1038/s41467-018-04419-x (2018).

8 Burgo, A. *et al.* Increased activity of the vesicular soluble N-ethylmaleimide-sensitive factor attachment protein receptor TI-VAMP/VAMP7 by tyrosine phosphorylation in the Longin domain. *The Journal of biological chemistry* **288**, 11960-11972, doi:10.1074/jbc.M112.415075 (2013).

9 Wen, W. *et al.* Lipid-Induced conformational switch controls fusion activity of longin domain SNARE Ykt6. *Molecular cell* **37**, 383-395, doi:10.1016/j.molcel.2010.01.024 (2010).

10 Blanchard, N. *et al.* TCR activation of human T cells induces the production of exosomes bearing the TCR/CD3/zeta complex. *J Immunol* **168**, 3235-3241 (2002).

REVIEWERS' COMMENTS:

Reviewer #1 (Remarks to the Author):

The authors have addressed my comments. They have come up with an acceptable conclusion regarding quantification of signals in fluorescence images. I have no further concerns.

Reviewer #2 (Remarks to the Author):

Tethering of vesicles to the Golgi by GMAP210 controls LAT delivery to the immune synapse

Zucchetti et al.
Revised Manuscript ID: NCOMMS-18-34369A

In the revised version of the manuscript, Zucchetti et al. have addressed my concerns adequately. They have included new data and text clearly demonstrating that the interaction between GMAP210 vesicles and LAT increases in stimulated cells consistent with their model. They have also included new imaging data showing the kinetic of GMAP210 recruitment to the immune synapse as coinciding with the later wave of LAT recruitment to the synapse. In addition, they have quantified the imaging and colocalization data as requested and they have also replaced the term “microclusters” with “puncta” to describe the imaged structures. They are unable to do the mitochondrial tethering assay in activated cells to show an increase in GMAP210 and VAMP7 interaction, but they clarify in their discussion that this transport pathway exists at steady state and is increased upon T cell activation. Overall, the new data and text included in the manuscript addresses my criticisms and strengthens their model.

Reviewer #3 (Remarks to the Author):

N/A

Point-by-point response:

To the reviewers

We would like to thank the reviewers for their positive comments. They did not raise further concerns.
